# Mutual Shaping of Circadian Body-Wide Synchronization by the Suprachiasmatic Nucleus and Circulating Steroids

**DOI:** 10.3389/fnbeh.2022.877256

**Published:** 2022-06-01

**Authors:** Yifan Yao, Rae Silver

**Affiliations:** ^1^Department of Psychology, Columbia University, New York City, NY, United States; ^2^Department of Neuroscience, Barnard College, New York City, NY, United States; ^3^Department of Psychology, Barnard College, New York City, NY, United States; ^4^Department of Pathology and Cell Biology, Graduate School, Columbia University Irving Medical Center, New York City, NY, United States

**Keywords:** steroid receptors, circadian rhythm, ultradian rhythm, androgen, estrogen, glucocorticoids

## Abstract

**Background:**

Steroids are lipid hormones that reach bodily tissues through the systemic circulation, and play a major role in reproduction, metabolism, and homeostasis. All of these functions and steroids themselves are under the regulation of the circadian timing system (CTS) and its cellular/molecular underpinnings. In health, cells throughout the body coordinate their daily activities to optimize responses to signals from the CTS and steroids. Misalignment of responses to these signals produces dysfunction and underlies many pathologies.

**Questions Addressed:**

To explore relationships between the CTS and circulating steroids, we examine the brain clock located in the suprachiasmatic nucleus (SCN), the daily fluctuations in plasma steroids, the mechanisms producing regularly recurring fluctuations, and the actions of steroids on their receptors within the SCN. The goal is to understand the relationship between temporal control of steroid secretion and how rhythmic changes in steroids impact the SCN, which in turn modulate behavior and physiology.

**Evidence Surveyed:**

The CTS is a multi-level organization producing recurrent feedback loops that operate on several time scales. We review the evidence showing that the CTS modulates the timing of secretions from the level of the hypothalamus to the steroidogenic gonadal and adrenal glands, and at specific sites within steroidogenic pathways. The SCN determines the timing of steroid hormones that then act on their cognate receptors within the brain clock. In addition, some compartments of the body-wide CTS are impacted by signals derived from food, stress, exercise etc. These in turn act on steroidogenesis to either align or misalign CTS oscillators. Finally this review provides a comprehensive exploration of the broad contribution of steroid receptors in the SCN and how these receptors in turn impact peripheral responses.

**Conclusion:**

The hypothesis emerging from the recognition of steroid receptors in the SCN is that mutual shaping of responses occurs between the brain clock and fluctuating plasma steroid levels.

***Comment on the Human Condition***

*Alexander Calder wrote in 1943 “[the artist] cannot see, or even conceive of a thing from all possible points of view simultaneously.” (Exhibit notes MOMA 2022).*

*To which we scientists add: The scientist cannot see a thing from all physical scales, at all times, from all points of view, simultaneously- but we keep trying.*

## Introduction and Background

### The Circadian Timing System

#### Multiple Levels of Circadian Timing

**FIGURE 1:**
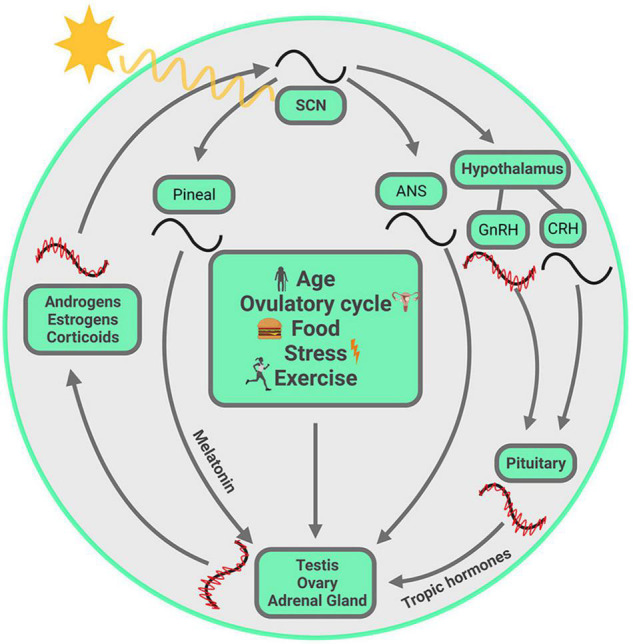
Visual summary of processes discussed in this review. The SCN, entrained by the ambient light, drives the daily variation of steroids from gonads and adrenal glands, chiefly but not exclusively through the hypothalamus-pituitary-axis. Within each gland, circadian clocks modulate steroid biosynthesis at several steps in the pathway, and these are unique to each gland. The steroids circulate in the systemic blood supply and reach the SCN where they act on their cognate receptors, thereby fine-tuning the SCN output. At multiple points, ultradian oscillation is necessary for circadian oscillation. These oscillations at different temporal scales are synchronized from one level to the next. Other entraining signals, such as age, ovulatory cycle, food, exercise and stress, may not act on SCN directly, but can modulate hormone synthesis from gonads and adrenals, with the consequence that circulating steroids reach the SCN and modulate its output. **Symbols:** red waves = ultradian oscillation, black waves = circadian oscillation. The ultradian rhythm of CRH is not included as it is not required for generating ACTH and CORT pulsatility (Walker et al., 2012). The ultradian oscillations are not drawn for SCN, melatonin and ANS as their ultradian rhythms are not covered in the current article.

The earth rotates on its axis and revolves around the sun, consequently providing a regularly recurring sequence of changes in the environment among which the daily alterations in the light and dark cycle are the most salient. To anticipate regularly recurring changes, and to optimally time behavioral and physiological responses, living organisms have evolved internal timing systems that measure durations of ∼24 h. For species survival, successful reproduction is obligatory, and success requires that the secretion of hormones necessary for breeding are aligned with optimal timing of ovulation, mating and seasonal changes. Steroids are lipid-derived hormones that play a key role in reproduction, metabolism, stress responses, homeostasis, and more. Systemically circulating steroid hormones and all these functions required for reproduction and survival are under the regulation of a circadian timing system (CTS) and its cellular/molecular underpinnings. The rhythms generated by this system prepare organisms for all manner of regularly recurring events in daily life, allowing predictive adaptation and appropriate coordination of bodily activities (Figure 1).

A key aspect of circadian rhythms is that they are endogenously organized and continue in the absence of external entraining cues. This is in contrast to diurnal rhythms that may also fluctuate on a daily basis but are sustained only in the presence of external synchronizing or driving cues. In the study of endocrine rhythms, as will become evident, it is often difficult to distinguish between circadian and diurnal rhythms.

#### Circadian Clocks Are Found in Cells and Tissues Throughout the Body

Since 1972 (Moore and Lenn, 1972; Stephan and Zucker, 1972), it has been known that the suprachiasmatic nucleus (SCN) is the site of a brain clock necessary for the expression of circadian rhythms. Since 1997 it was known that the individual neurons of the SCN themselves contain molecular elements that constitute a 24-h clock (Sun et al., 1997; Tei et al., 1997). With that background, one of the most surprising findings in the field in its time was the discovery that the cellular-molecular clock mechanisms are found not only in the brain but also in virtually all the cells of the body (Balsalobre et al., 1998). Body-wide, multi-level synchronization is achieved by coordinating activity of these ubiquitous cellular/molecular clocks.

**FIGURE 2:**
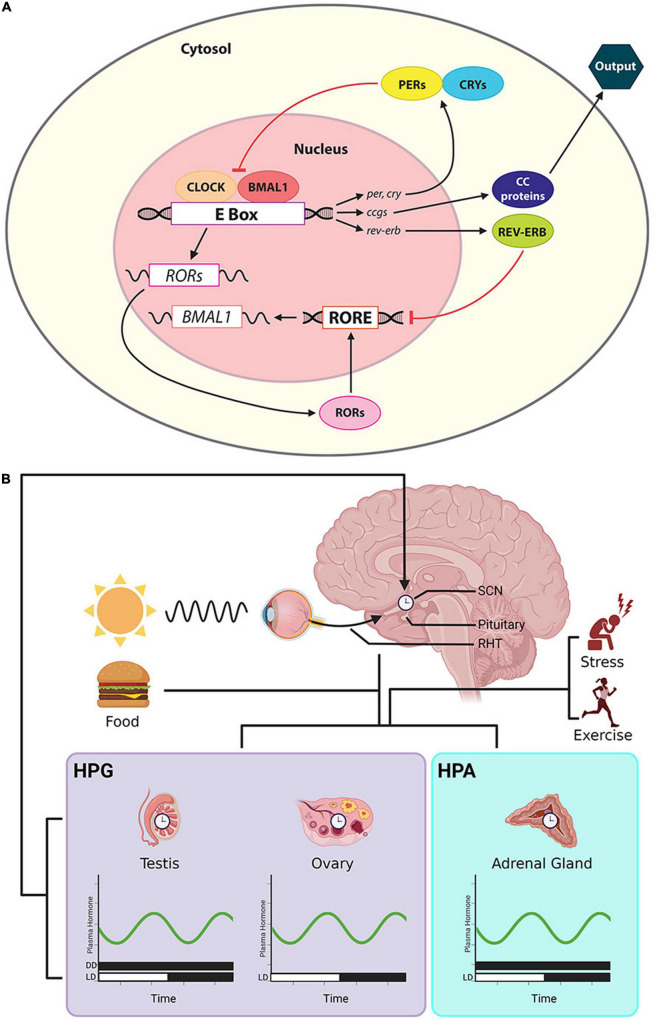
Cellular/molecular clock, the CTS and rhythmic steroid production. **(A)** The cellular clock involves a primary loop controls the formation of dimer CLOCK/BMAL1. These proteins are translocated to the E-box of the promoter of *per* and *cry*, activating their transcription. PER and CRY proteins in turn suppress the translation of CLOCK/BMAL1. Orphan nuclear receptor genes, Rev-Erbα/β and RORα/β, form a secondary transcriptional loop. REV-ERB and ROR protein compete for RORE at the promoter region of bmal1. REV-ERB suppresses bmal1 transcription and ROR activates it. The activities of these clock genes further regulate downstream ccgs and their protein products. **(B)** Output from the master clock in the SCN sends timing information to peripheral clocks in the gonads and adrenal glands, shaping the timing of daily secretion of steroids. The steroids circulate in the systemic blood supply, reaching cells throughout the body. Afferent input to the SCN includes photic cues that travel via the RHT and cues that derive from changing hormone secretions that are a consequence of factors such as stress, exercise and eating. **Symbols:** the bars along the abscissa represent the time of day in terms of a 24-h clock. Black bar denotes dark, and the white bar indicate a period of light. For some hormones, measures have been made in both DD and LD cycles.

Circadian clock mechanisms have been extensively studied (reviewed in Takahashi, 2016). Briefly, transcriptional-translational loops involve a number of key clock genes and proteins. The primary loop starts with dimer CLOCK/BMAL1. These proteins are translocated to the E-box of the promoter regions of the clock genes, *per* and *cry*, and activate their transcription. PER and CRY proteins in turn suppress the translational activity of CLOCK/BMAL1 proteins. Additionally, members of the orphan nuclear receptor genes, *Rev-Erbα/β and RORα/β*, form a secondary transcriptional loop. REV-ERB and ROR protein compete for retinoic acid-related orphan receptor response element (RORE) sites at the promoter region of *bmal1*. The former suppresses *bmal1* transcription and the latter activates it (Guillaumond et al., 2005). These two transcriptional-translational negative feedback loops form the basis of daily oscillation at the molecular level (Figure 2A).

It is estimated that about 10% of genes exhibit circadian rhythms in a given tissue, but clock-controlled genes (ccgs) differ considerably among tissues (Panda et al., 2002). In mice, 43% of all protein coding genes showed circadian rhythms in transcription in at least one organ of twelve organs studied (Zhang et al., 2014). At the level of the whole organism, circadian oscillation is manifest in daily physiological and behavioral rhythms, including energy metabolism (Koronowski and Sassone-Corsi, 2021), circulating hormones (Roa et al., 2017), body temperature (van der Vinne et al., 2018), and sleep–wake cycles (Lee et al., 2018) and determine responses to drug administration, impacting treatment outcomes (Tamai et al., 2018). These cell-based clocks, ccgs and their protein products are ubiquitous: given their presence in the most organs and tissues of the body, we explore how circulating steroid hormones that reach all these sites contribute to the acquisition of information about time of day.

### The Present Goal

The present goal is to explore how the SCN influences rhythms of circulating steroid secretion, and how synchronization and de-synchronization of the CTS impact and are impacted by steroid hormone actions on cellular/molecular clocks located throughout the body, including those of the SCN. We explore plasma levels, inter-individual variability, differences among species and between the sexes. Rhythmic response patterns are locally unique. A great number of studies have examined oscillation in one brain nucleus or one bodily tissue at a time and the challenge is to understand the system as a whole.

The CTS presents an empirically accessible system for studying coordination of signals from the environment and from within the body. The molecular, cell-based clock mechanism can be impacted by external and internal stimuli in a number of ways, thereby producing alignment or misalignment of temporal changes in the body. To understand how coordination of bodily clocks is achieved, we try to capture simultaneously ongoing events at several temporal scales, from ultradian to circadian to menstrual/ovulatory to seasonal, and at different levels of analysis from cells to organs to body-wide events. The cartoon in Figure 1 is a visual representation at a glance of all the elements at play in the CTS. The visual summary allows us to highlight the idea that the CTS is not fundamentally hierarchically organized, and that one could begin a discussion at any starting point in the circle. The image also conveys the idea that everything depicted happens simultaneously. Finally, it describes the sequence of topics covered in this review, highlighting the key elements of the storyline which begin and end with the SCN.

Circulating steroid hormones of both gonadal and adrenal origin fluctuate daily, and act on tissues throughout the body. Daily hormone rhythms, however, are not fixed characteristics but are modulated by various behavioral, physiological and environmental conditions (Figure 2B). As an example, steroids affect level of physical activity (reviewed in Vitale et al., 2018) and physical activity in turn can improve muscle function, modulate body weight, and reduce feelings of depression. In contrast, physical inactivity is associated with increased incidence of cancer, obesity, diabetes, etc. Examination of the entire system impacted by circulating steroid hormones, reveals how the timing systems of the body regulate local and global responses and are themselves regulated.

## Suprachiasmatic Nucleus as a Master Clock

Optimal synchronization of oscillators in the CTS is achieved by multiple feedback loops in this system (Figure 2). Clearly, in a loop there is no “top” of a hierarchy. It is convenient nevertheless to view the hypothalamic SCN as a starting point for describing the organization of the CTS and to consider it as the master brain circadian clock.

The SCN is a self-sustained oscillator, capable of expressing very long-term circadian rhythms, even in the absence of cues from the environment, and even when isolated from the brain and placed in a tissue culture preparation (Yamazaki et al., 2000). This indicates that SCN circadian oscillation does not require input from the environment or from the rest of the brain or body. The SCN itself is made up of ∼20,000 neurons (in rodents) arranged into two main subregions, termed core and shell (Silver and Moore, 1998; Abrahamson and Moore, 2001; Lokshin et al., 2015). The core is delineated by vasoactive intestinal polypeptide (VIP)-expressing neurons and is the major site of retinal terminals from the retinohypothalamic tract (RHT) (Lokshin et al., 2015). Neurons of the core have relatively low amplitude oscillation of circadian clock genes and proteins while the shell is largely delineated by arginine vasopressin (AVP)-expressing neurons and has high amplitude oscillation (Hamada et al., 2004).

Within the core and shell regions, there are several additional clusters of various peptidergic cell types (Silver and Moore, 1998; Abrahamson and Moore, 2001). While there is substantial evidence that neurons of the core and shell have different functions, the role of these clusters of peptidergic cells other than AVP and VIP is not well understood and not much explored. One possibility is that they respond to non-photic cues and reflect species-specific adaptations. This consideration comes up again at the end of this review, when we consider sites of action of steroids in the SCN.

The special role of the SCN is attributable to its unique role in synchronizing the body to the light:dark (LD) cycle of the local environment via specialized non-image forming photic inputs from the retina (Moore and Lenn, 1972). The SCN is a component of a unique sensory processing system with specialized photoreceptors – retinal melanopsin-containing photosensitive ganglion cells (Berson, 2003). The photic information that functions to synchronize the SCN’s circadian rhythms to the local environment is processed in a specialized manner, different from that of the image forming visual system. Unlike the image-forming visual system which functions equally well at all times of day if conditions are constant, photic cues are effective in resetting SCN oscillations only at specific times of day (Kornhauser et al., 1990). Also, different from the image-forming visual system, photic input is integrated over relatively long durations (Nelson and Takahashi, 1991) and the authors suggested that this property is important in rendering the SCN unresponsive to environmental ‘noise’ that could interfere with entrainment to regularly recurring LD cycles.

During daytime, transient photic cues, such as cloud cover or movement from shade to sunshine in nature, do not reset the SCN. Regularly recurring photic cues do reset the clock. In addition, the SCN responds to constant illumination (Rumanova et al., 2020). A question considered in this review is whether the SCN has parallel specialized responses, namely to transient, regularly recurring and steady state cues to the afferent signals presented by circulating steroids.

### Diffusible Outputs

On the output side, the SCN sends temporal information to the rest of the brain and body via both neural efferents (Morin, 2013) and by diffusible signals (Lesauter and Silver, 1998; Li et al., 2012). The timing signal from the SCN to hypothalamic neuroendocrine cells is apparently not continuously available, but appears to be communicated at a specific time of day, namely at the transition from light to dark measured by the onset of FOS expression (Butler et al., 2012). Interestingly, transplant studies of the SCN indicate that the circadian signal for hormone secretion is different from that regulating circadian locomotor behavior (Meyer-Bernstein et al., 1999). SCN transplants restore rhythms of locomotor activity, but not rhythmic endocrine secretion, indicating that they are based on different output signals. One possible explanation is that the latter require neural efferents while the former do not. Another possibility is that signals of SCN origin travel to their target sites via a portal system that courses between the SCN and the organum vasculosum of the lamina terminalis, a circumventricular organ lying in the 3rd ventricle (Yao et al., 2021). From the cerebral spinal fluid humoral signals can reach large volumes of parenchyma.

### Neural Efferents to Endocrine Systems

Suprachiasmatic nucleus control of steroid rhythms is achieved through actions on tropic hormones, on pineal melatonin, and via the autonomic nervous system (ANS). The major neural efferents of the SCN to neuroendocrine systems include both mono- and multi-synaptic connections. Efferents of SCN VIP neurons that lie in the core region of the SCN synapse monosynaptically onto hypothalamic gonadotropin releasing hormone (GnRH) neurons (van der Beek et al., 1993; Van Der Beek et al., 1997). The density of innervation differs between the sexes and increases during puberty, associated with maturation of the hypothalamic-pituitary-gonadal axis (HPG) (Horvath et al., 1998; Kriegsfeld et al., 2002). VIP_2_ receptors are localized in the GnRH neurons (Smith et al., 2000). In brain slices, GnRH neurons from both female and male mice are excited by VIP through its receptor-mediated signaling, measured by increased firing rate and intracellular calcium (Piet et al., 2016). This effect was independent of stage of the estrous cycle and the time of day that the slices had been collected. In a multisynaptic pathway to releasing hormones, efferents of AVP neurons of the SCN shell synapse directly onto hypothalamic kisspeptin (Kp) neurons which in turn contact GnRH neurons (reviewed in Simonneaux, 2020). The SCN also sends monosynaptic efferents to neurons in the paraventricular nucleus. These neurons are thought to participate in balancing the sympathetic and parasympathetic systems to many organs including the pineal, gonads and adrenal glands (Buijs et al., 2019). Both routes regulate the glandular cellular clocks modulating steroid biosynthesis, discussed below.

Another major neural pathway through which SCN efferents regulate hormone secretions is by way of actions on the pineal gland through a multisynaptic innervation via the superior cervical ganglion. There is a vast literature on pineal melatonin, developed ever since it was determined that the duration of pineal melatonin secretion encodes the duration of the night, which of course becomes progressively longer during fall and winter and shorter during the spring and summer. Systemically circulating melatonin acts on multiple sites including steroidogenic cells in testes, ovaries, and adrenal glands that ultimately affect plasma steroid levels. The literature on the functions and mechanisms of action of melatonin is beyond the scope of this review and the reader is directed to Pfeffer et al. (2018), Cipolla-Neto et al. (2021), and Reiter and Sharma (2021).

## Ultradian Frequencies Underlie Circadian Hormone Rhythms

As noted above, the SCN entrains and modulates rhythms of circulating steroids by multiple routes. These changes occur at several spatial levels (cells, organs) and temporal scales (ultradian, circadian). Higher frequency ultradian rhythms in hormone secretion underlie the circadian rhythms, and both occur in concert.

### Hypothalamic-Pituitary-Gonadal Axis

The occurrence of a daily rhythm underlying the ovulatory luteinizing hormone (LH) surge and more broadly, the ovulatory cycle, has been known for decades (Everett and Sawyer, 1949). Unraveling the mechanisms underlying this daily event began in the early 2000s, with the discovery of ultradian “*pulses*” and circadian “*surges*” (Clarkson et al., 2017; Simonneaux, 2020; Nagae et al., 2021; Uenoyama et al., 2021). Pulsatility of GnRH is necessary for the release of pituitary gonadotropic hormones (Belchetz et al., 1978; Knobil, 1980; Urbanski et al., 1988) and involves a mechanism termed the “GnRH pulse generator.” Pulsatile GnRH secretion is produced by a population of hypothalamic Kp neurons of the arcuate nucleus (ARC). These cells generate synchronized GnRH release about every 90 min, driving pulsatile pituitary gonadotropin secretion. Pulsatility in plasma gonadal steroid levels is temporally linked to pulsatile pituitary gonadotropin.

The frequency of GnRH pulsatility determines the proportion of LH versus follicle stimulating hormone (FSH) released such that low frequency pulsatility of GnRH favors FSH synthesis whereas high frequency pulsatility facilitates LH (Wildt et al., 1981). In both sexes, testosterone (T) and progesterone (P) pulses occur ∼10–40 min after the peak of the LH pulse. Estrogens follow closely or are concurrent with T and P pulses. In women, the episodic secretion of LH and estradiol (E2) is correlated with systematic changes across the menstrual cycle. The inter-pulse intervals of LH and E2 steadily decrease over the follicular phase, and significantly increase during the luteal phase (Bäckström et al., 1982). The frequency of GnRH pulses, however, do not increase during ovulation, indicating that a mechanism other than the pulse generator underlies the ovulatory LH surge (Adams et al., 1994).

**FIGURE 3:**
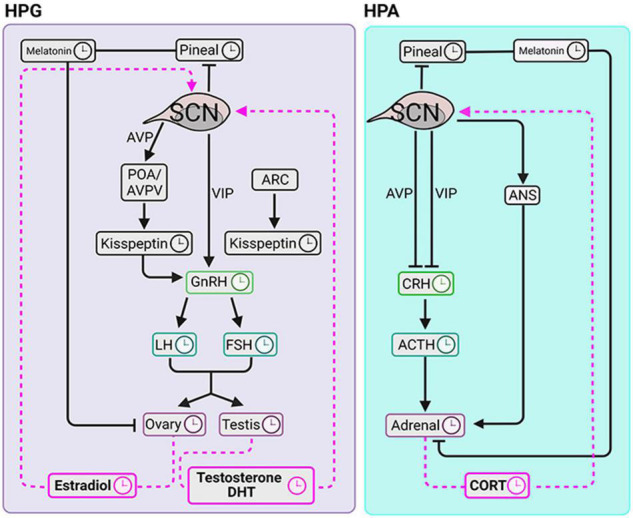
Circadian clocks of the HPG and HPA axes. The SCN coordinates diurnal fluctuations in the HPG **(left panel)** and HPA **(right panel)** axes thereby impacting steroidogenesis. The signals to the gonads travel via the hypothalamus-pituitary-axis and via pineal melatonin. SCN signals to the adrenal glands course via hypothalamus-pituitary-axis, pineal melatonin and via autonomic nervous system. **Symbols:** clockfaces indicate sites that express daily rhythms. Black lines are pathways from the SCN to the downstream elements. Pink dashed lines indicate signaling pathways from circulating glandular steroids to the SCN.

The aforementioned pulsatility of gonadal trophic hormones and ovarian hormones is regulated by the SCN. By integrating information about hormonal pulses, the SCN becomes the key to timing the ovulatory surge in LH. Environmental light cues synchronize the timing of AVP and VIP release from the SCN. In the late afternoon, under the condition of high circulating E2, VIP directly activates GnRH neurons. Vasopressin produces positive feedback on Kp neurons located in the POA/AVPV in the rostral hypothalamic regions (reviewed in Matsuda et al., 2019). In addition, the ARC Kp neurons in the preoptic area (POA)/anteroventral periventricular nucleus (AVPV) produce GnRH pulsatility in both males and females while only in females Kp neurons of the POA/AVPV region mediate the preovulatory GnRH/LH surge. Thus, anatomically distinct hypothalamic regions and neuropeptide projection pathways are critical to the timing of the LH surge and GnRH pulse generator (Figure 3).

### Hypothalamic-Pituitary-Adrenal Axis

As in the HPG axis, pulsatility is required for rhythmic corticosteroid secretion. Specifically, adrenocorticotropic hormone (ACTH) pulses are indispensable for generating pulsatile glucocorticoids (Spiga et al., 2011). In contrast to the HPG axis however, the pulse generator of the corticosteroids seems to be downstream of the hypothalamus. This conclusion derives from evidence that in the HPA axis, at an optimal concentration, a constant level of corticotropin releasing hormone (CRH) can generate pulsatile secretion of the ACTH and CORT (Walker et al., 2010, 2012). This suggests that the pituitary and adrenal glands form a closed feedback loop capable of fine tuning the ultradian oscillations of ACTH and glucocorticoids.

The SCN is not part of the HPA pulse generator; however, it plays a role in modifying the number of CORT pulses thereby producing the daily variation of this hormone (Waite et al., 2012). Specifically, SCN lesions cause an increase in the number of CORT pulses during the light period and this change abolished the diurnal rhythm of circulating CORT. This result indicated the SCN exert an inhibitory effect on the HPA pulse generator during the (behaviorally) inactive phase of LD cycle.

In summary, in both the HPG and HPA, ultradian pulses occur at multiple levels and contribute to the higher order diurnal and circadian oscillation rhythms. In one attempt to conceptualize the relationship between short and long term oscillations, a coupled oscillator model that assumed variable-strength coupling within and among hormonal responses was proposed (Grant et al., 2018). In this model various temporal rhythms of the brain and periphery could be linked by a deeper understanding of coupling mechanisms.

## Circulating Steroids and Their Fluctuations

### Circulating Steroids Have Multiple Glandular Sources

The ovaries, testes and adrenal glands are differentially modulated by the CTS, thus in order to understand which CTS signals affect particular steroid levels, it is important to know its source(s). Specific circulating steroids may originate from one or more sources (summarized in Table 1 for male–female sex steroids; as circulating corticoids are almost exclusively produced by adrenal cortex, they are not included in the table). The source of a steroid can be determined by assays performed before and after ovariectomy (OVX), orchidectomy or adrenalectomy (ADX), and in some cases, following adrenal hormone suppression by the potent synthetic glucocorticoid, dexamethasone treatment. Most data on daily and circadian variation of circulating steroids are available for humans and rodents, specifically rats and mice. (Though there is lovely data on other species, including Siberian and Syrian hamsters, this work is beyond the scope of the present review). Importantly, there are two major differences in sex steroid metabolism between humans and rodents (specifically rats and house mice). In humans, but not in these rodents, serum sex steroids are bound to high affinity steroid hormone binding globulin (Jänne et al., 1998). Second, in humans but not rats and house mice, the adrenal glands secrete substantial amounts of C-19 androgens, precursors of dehydroepiandrosterone (DHEA) and androstenedione (Δ4) (van Weerden et al., 1992; Boonstra et al., 2008; Soma et al., 2015; Quinn et al., 2016).

**TABLE 1.1:** Glandular sources of sex steroids in men.

		Adrenal	Testis	References
Androgens	T	7%	90–93%	Scott et al., 1980; Bélanger et al., 1989
	DHT	0–33%	66–100%	Meikle et al., 1979; Bélanger et al., 1989
	Δ4	50%	14–50%	Vermeulen and Verdonck, 1976; Stege et al., 1987; Georgiadis et al., 1992
	DHEA	90%	10%	Vermeulen and Verdonck, 1976
	DHEAS	83–100%	0–17%	Vermeulen and Verdonck, 1976; Stege et al., 1987; Sharifi and Auchus, 2012
Estrogens	E1	50–60%	35–54%	Saez et al., 1972; Arlt et al., 1998; de Ronde et al., 2005
	E2	0	100%	Longcope et al., 1969, 1978; Kelch et al., 1972; Saez et al., 1972; Russell and Grossmann, 2019
Progestin	P	100%	0	Abraham et al., 1971; Vermeulen and Verdonck, 1976

*DHEA, dehydroepiandrosterone; DHEAS, dehydroepiandrosterone sulfate; DHT, dihydrotestosterone; E1, estrone; E2, estradiol; P, progesterone; T, testosterone; Δ4, androstenedione.*

**TABLE 1.2:** Glandular sources of sex steroids in women.

		Adrenal gland				Ovary				References
		Menstrual cycle			Post- menopause	Menstrual cycle			Post- menopause	
		Follicular	Midcycle	Luteal		Follicular	Midcycle	Luteal		
Androgens	T	66%	40%	66%	50–100%	33%	60%	33%	21–50%	Abraham, 1974; Vermeulen, 1976; Couzinet et al., 2001; Fogle et al., 2007; Labrie, 2011
	DHT	50%			100%	50%			27%	Vermeulen, 1976; Labrie, 2011
	Δ4	30%-55%	30%	40%	66%-100%	45%	70%	60%	10%-33%	Abraham, 1974; Vermeulen, 1976; Arlt et al., 1998; Couzinet et al., 2001; Fogle et al., 2007; Labrie, 2011
	DHEA	80%			56%	20%			18%	Abraham, 1974; Vermeulen, 1976; Labrie, 2011
	DHEAS	96%	90%	96%	100%	4%	10%	4%	19%	Abraham, 1974; Vermeulen, 1976; Couzinet et al., 2001; Labrie, 2011
Estrogens	E1	50%-60%			100%	100%			0	Saez et al., 1972; Kim et al., 1974; Arlt et al., 1998; Labrie, 2011
	E2	0			50%-83%	100%			17–50%	Saez et al., 1972; Kim et al., 1974; Arlt et al., 1998; Labrie, 2011
Progestin	P	0			100%	100%			0	Kim et al., 1974; Vermeulen, 1976

*DHEA, dehydroepiandrosterone; DHEAS, dehydroepiandrosterone sulfate; DHT, dihydrotestosterone; E1, estrone; E2, estradiol; P, progesterone; T, testosterone; Δ4, androstenedione.*

### Circulating Steroids Have Daily Fluctuations

Studies of circulating hormones have produced a confusing picture of daily changes. Part of the problem is attributable to methodological constraints. Episodic steroid hormone secretion and diurnal variation in serum levels makes the interpretation of a single value uninterpretable. In animal studies, the problems of drawing blood samples in small rodents, a stressful procedure, is compounded by the difficulties in collecting sufficient material for serial samples within individuals (Yoo and Napoli, 2019). A source of differences among study results is the interval after an experimental manipulation that the assay is performed the pre- and post- menopause or andropause state, and in females, the stage of menstrual/ovulatory cycle. Pre-analytic methodological factors can also be a source of different results among studies (Gholib et al., 2021). In work with humans the importance of accurate steroid level measurements in diagnosis of diseases such as adrenogenital syndrome, precocious and delayed puberty hypercortisolism and adrenal insufficiency stimulated the development of more accurate and sensitive assays and improved sample collection methods. Newer, non-invasive methods involve fecal, urinary or salivary sampling, along with improvements in assay detection methods (discussed in Nilsson et al., 2015; Conklin and Knezevic, 2020) and the possibility of measuring numerous steroids in a single sample (Hill et al., 2019) have improved the quality of information on daily rhythms. Finally, adding to the difficulty of interpreting the results is that a great many different experimental designs have been used in studies of hormone levels with a variety of photic conditions, sampling times, sampling intervals, and sample sizes (summarized in Table 2).

**TABLE 2:** Experimental design in studies of daily hormone rhythms.

		Circulating hormone	Species	Sex	LD or DD	Light duration	# of Subjects	Sampling interval (hrs)	Repeated measures	References
Sex hormones	Androgens	T	Human	M	LD	16	10	0.25–0.5	Yes	Leproult and Van Cauter, 2011
				M	LD	16	5	0.5–0.75	Yes	Cooke et al., 1993
				M	LD	16	5	4	Yes	Reinberg et al., 1975
				M	LD	17	3	1	Yes	Guignard et al., 1980
				M	LD	16	6	1	Yes	Juneja et al., 1991
				M	LD	16	10	4	Yes	Opstad, 1994
				M	LD	16	20	0.16	Yes	Spratt et al., 1988
				M	LD	?	12–17	4	Yes	Bremner et al., 1983
				F	LD	?	16	3	Yes	Ostrowska et al., 1998
				M+F	LD	?	173–264	Various	Partial	Dabbs, 1990
				M+F	LD	17	17	4	Yes	van Kerkhof et al., 2015
				M+F	LD	?	32	4	Yes	Nicolau et al., 1984
			Rats	M	LD	14	56	4	No	Wong et al., 1983
				M	LD	14	7	0.16	Yes	Waite et al., 2009
				M	LD	14	6	2–3	No	Kalra and Kalra, 1977
			Mice	M	LD	12	64	2	No	Mock et al., 1978
				M	LD	12	114–132	4	No	Lucas and Eleftheriou, 1980
				M	DD	N/A	72	2	No	Mock et al., 1978
				M+F	LD	12	16	2	Yes	Auer et al., 2020
		DHT	Human	M	LD	17	3	1	Yes	Guignard et al., 1980
			Rats	M	LD	14	6	2–3	No	Kalra and Kalra, 1977
		Δ4	Human	M	LD	16	10	4	Yes	Opstad, 1994
				M	LD	?	6	0.5	Yes	Goldman et al., 1985
				M	LD	17	3	1	Yes	Guignard et al., 1980
		DHEA	Human	M	LD	17	3	1	Yes	Guignard et al., 1980
				M	LD	16	10	4	Yes	Opstad, 1994
				F+FPM	LD	16	7	0.25	Yes	Liu et al., 1990
				F	LD	?	10	0.5	Yes	Carlström et al., 2002
				M+F	LD	15	10	2–4	Yes	Ceresini et al., 2000
		DHEAS	Human	M	LD	16	8	1–2	Yes	Zhao et al., 2003
				M	LD	16	10	4	Yes	Opstad, 1994
				M	LD	17	3	1	Yes	Guignard et al., 1980
				F	LD	?	16	3	Yes	Ostrowska et al., 1998
				F	LD	?	10	0.5	Yes	Carlström et al., 2002
				F	LD	16	15	2	Yes	Carandente et al., 1990
				M+F	LD	17	17	4	Yes	van Kerkhof et al., 2015
				M+F	LD	?	32	4	Yes	Nicolau et al., 1984
	Estrogens	E1	Human	M	LD	16	4	0.5	Yes	Leymarie et al., 1974
				Fl	LD	?	11	0.5–1	Yes	Patrick et al., 1979
		E2	Human	M	LD	16	4	0.5	Yes	Leymarie et al., 1974
				M	LD	16	6	1	Yes	Juneja et al., 1991
				F	LD		17	1	Yes	Rahman et al., 2019
				FPP	LD	16	7	0.3	Yes	Boyar et al., 1976
				F	LD	16	15	2	Yes	Bao et al., 2003
				F	LD	16.5	15	?	Yes	Carandente et al., 1989
				FPP	LD	?	11	0.5–1	Yes	Patrick et al., 1979
				M+F	LD	17	17	4	Yes	van Kerkhof et al., 2015
				M+F	LD	?	32	4	Yes	Nicolau et al., 1984
			Rats	F	LD	14	5–21	Various	No	Kalra and Kalra, 1974
		E3	Human	Fl	LD	?	11	0.5–1	Yes	Patrick et al., 1979
	Progestin	P	Human	M	LD	16	10	4	Yes	Opstad, 1994
				F	LD	0	17	1	Yes	Rahman et al., 2019
				F	LD	13	7	0.16	Yes	Veldhuis et al., 1988
				F	LD	16.5	15	?	Yes	Carandente et al., 1989
				F	LD	17	6	0.25	Yes	Kottler et al., 1989
				M+F	LD	17	17	4	Yes	van Kerkhof et al., 2015
				M+F	LD	?	32	4	Yes	Nicolau et al., 1984
			Rats	M	LD	14	6	2–3	No	Kalra and Kalra, 1977
				M	LD	8	6	1–4	Yes	Hueston and Deak, 2014
				F	LD	14	5–21	Various	No	Kalra and Kalra, 1974
				FP	LD	12	42–48	3	No	Crew et al., 2016
			Mice	F	LD	13	50	4	Yes	Bailey, 1987
Corticoids	Gluco-	Cortisol	Human	M	LD	16	10	4	Yes	Opstad, 1994
				M	LD	17	12	3	Yes	Sjöberg et al., 1979
				M	LD	17	3	1	Yes	Guignard et al., 1980
				F	LD	?	10	0.5	Yes	Carlström et al., 2002
				F	LD	16	15	2	Yes	Carandente et al., 1990
				F+FPP	LD	16	7	0.25	Yes	Liu et al., 1990
				M+F	LD	16	10	2–6	Yes	Cugini et al., 1992
				M+F	LD	15.5	10	4	Yes	Portaluppi et al., 1990
				M+F	LD	?	45	<0.5	Yes	Van Cauter et al., 1996
				M+F	LD	?	32	4	Yes	Nicolau et al., 1984
		Corticosterone	Rats	M	LD	12	6	3	No	Hilfenhaus, 1976
				M	LD	8	6	1–4	Yes	Hueston and Deak, 2014
			Mice	F	LD	14	200	4	No	Nichols and Chevins, 1981
				M	LD	12	24–36	4	No	Oster et al., 2006b
				M	DD	N/A	24–36	4	No	Oster et al., 2006b
	Mineralo-	Aldosterone	Human	M	LD	15	4	1	Yes	Ryoyu et al., 1984
				M+F	LD	16	10	2–6	Yes	Cugini et al., 1992
				M+F	LD	15.5	10	4	Yes	Portaluppi et al., 1990
				M+F	LD	?	32	4	Yes	Nicolau et al., 1984
			Rats	M	LD	12	6	3	No	Hilfenhaus, 1976

*M, male; F, female; FP, pregnant; FPM, postmenopausal; FPP, pre-puberty; N/A, not applicable; ?, not reported. All samples are collected over an interval of at least 24 h.*

#### Sex Hormones

For convenience of communication, we divide steroid hormones into the following five groups: androgens, estrogens, progestogens, glucocorticoids, and mineralocorticoids (Miller, 1988). Based on their sites of synthesis and their functionality, the first three steroids are often termed sex hormones and the remaining two are termed corticoids, though a particular steroid may be produced by more than one gland. The sex hormones, namely androgens, estrogens, P are considered in turn.

##### Androgens

The androgenic hormones found in plasma include T, dihydrotestosterone (DHT) and the pro-androgens – dehydroepiandrosterone sulfate (DHEAS), DHEA, Δ4. Of these, T is one of the most intensively studied hormones, likely as it has been much touted as the basis of sex differences in behavior and abilities, a claim with substantial social and political consequences (Herbert, 2015).

###### T and Dihydrotestosterone

In rodents, the experimental literature on circulating androgens presents a seriously confusing picture of daily and circadian fluctuations in plasma androgens. In animals maintained in stable LD cycles, there are reports of monomodal, bimodal and trimodal patterns. For example, in Charles River rats held under a 14:10 LD cycle and sampled at intervals of 2–3 h, plasma T had a monomodal pattern with a peak ∼2.5 h before lights off and a trough at 2.5 h after lights off (Kalra and Kalra, 1977). In the latter report, the daily pattern of changes in plasma DHT was similar to that of T. In contrast, male Sprague-Dawley rats held under a 14:10 LD cycle (lights-on 5:00–19:00 h), and sampled at 10 min intervals, had bimodal plasma T peaks at about the mid-point of the day (∼12:30 h) and about the mid-point of the night (∼00:50 h, Waite et al., 2009). Bimodal peaks were also reported in mice sampled every 4 h, but with peak time differences between BALB/cBy and C57BL/6By strains (Lucas and Eleftheriou, 1980). As a final example, in Sprague-Dawley rats held under 12:12 LD cycle and sampled at several different intervals in a series of thirteen studies, plasma T showed a trimodal pattern (Mock et al., 1978). This trimodal pattern was maintained under conditions of constant darkness (DD), suggesting circadian control, and it was of higher amplitude in DD than in constant light. The latter authors also describe seasonal changes. In an attempt to clarify the basis of the differences among studies Wong and their collaborators (see Figure 4 in Wong et al., 1983) examined the contribution of age, strain and season on gonadal hormone levels in rat serum at 4 h intervals holding constant the laboratory conditions and housing conditions (LD 14:10, lights on at 05:00 h). The results point to marked differences in diurnal rhythms of T in the three strains of rats at the various ages studied, with evidence of uni-, bi-, and trimodal peaks.

Comparison of the former results to work using non-invasive procedures provide another window on the complex pattern of diurnal androgen levels. Auer et al. (2020) examined T metabolites in fecal and urinary samples to examine diurnal fluctuations in male and female mice. Males excreted significantly more radiolabeled fecal T metabolites than did females (59% vs. 49.5% respectively) and formed different metabolites. T metabolite excretion patterns were affected by the time of łH-testosterone injection. Males but not females showed fluctuations in daily T metabolite levels with the highest metabolite concentrations in the early night (∼21:00 h) and in the mid-nighttime active period (15:00 – 17:00 h, LD 12:12, lights on at 08:00 h). The results of the enzyme immunoassay used here were supported by showing that human chorionic gonadotropin (hCG) administration increased, whereas castration reduced levels of T metabolites. After taking into account intestinal gut passage time, the authors estimated that T peaks toward the end of the dark phase when mice are most active and around noon, in the middle of the light phase.

In humans, highly controlled experimental conditions and high frequency sampling methods clarify patterns of T secretion and can help to understand sources of differences among studies. Much of the evidence points to an androgen peak around wakeup time and early morning. Total and free plasma T on average have daily variation in men and women, peaking in the morning, declining throughout the day, and reaching a nadir in the late night (Guignard et al., 1980; Bremner et al., 1983; Khan-Dawood et al., 1984; Dabbs, 1990; Ostrowska et al., 1998). But the averages can obscure individual differences. In a study performed with 20 min blood sampling intervals, in some but not all men, a diurnal pattern of T secretion with a decline in T levels from morning to evening was seen, pointing to individual differences (Spratt et al., 1988). In a particularly fine-grained analysis of free and bound T, in which five men were sampled every 30–45 min for 25.5 h, a dominant median peak of T was observed at ∼05:30 h, with a sleep time of ∼midnight – 08:00 h (Cooke et al., 1993). Consistent with these observations, there were two lesser peaks during the wake hours and individual differences were also seen in this small group. A similar peak time of T was reported in a sample of ten men, with sleep deprivation producing a decline of ∼15% in T levels (Leproult and Van Cauter, 2011). Finally, there is evidence that the daily peak time of plasma T changes seasonally, as suggested in the rodent work discussed above (Reinberg et al., 1975; Smith et al., 2013). Rhythmicity in the other androgenic steroids has also been studied extensively.

###### Androstenedione

Δ4 is synthesized in the testes and ovaries as well as in the adrenal glands. In females, Δ4 is metabolized to provide around half of all T and almost all of the body’s estrone (E1). Plasma levels of Δ4, like T, peak in the early morning (Guignard et al., 1980; Goldman et al., 1985; Opstad, 1994). Salivary Δ4 has been used as an index of androgen production: In a population of prepubertal girls being treated for congenital adrenal hyperplasia plasma Δ4 and plasma concentrations T were correlated, as were salivary Δ4 and plasma T concentrations (Young et al., 1988).

###### Dehydroepiandrosterone

Circulating DHEA, derives from both gonadal and adrenal glands, and in most studies, like T, it peaks in the morning, gradually decreases throughout the day, and then increases again before awakening (Sirinathsinghji and Mills, 1985; Ceresini et al., 2000). As might be expected, due to declining gonadal sources, the plasma concentration of DHEA decreases in elderly men and postmenopausal women (Liu et al., 1990; Ahn et al., 2007).

###### Dehydroepiandrosterone Sulfate

In contrast to T, which is of both gonadal and adrenal origin, DHEAS derives exclusively from the adrenal glands in both men and women and diurnal changes in DHEAS are readily discernible. Plasma DHEAS levels peak in the late morning or early afternoon and reach a nadir during late night (Sirinathsinghji and Mills, 1985; Carandente et al., 1990; Opstad, 1994; Ostrowska et al., 1998; Zhao et al., 2003). As for cortisol (CORT, the major glucocorticoid in humans), there is an increase in DHEAS after morning awakening (Ghiciuc et al., 2011). There has been substantial interest in using DHEAS as a marker for understanding the contribution of adrenal hormone secretion to daily plasma levels of various steroids as this information is important in the assessment of androgen status in women and prepubertal children. DHEAS is strongly bound to serum albumin and has a very low metabolic clearance rate. To assess the usefulness of this as a marker, serum concentrations of DHEA, DHEAS, CORT and albumin were sampled every half hour over a 24 h period in 10 women (Carlström et al., 2002). The results indicate that daily fluctuations in serum DHEAS are related to alterations in its main serum albumin binding protein, rather than to changes in adrenocortical steroid secretion. The influence of the menstrual cycle on the rhythmicity of DHEAS is negligible (Carandente et al., 1990). With age however, plasma concentration of DHEAS decreases and the circadian rhythm becomes blunted in both men and women (Nicolau et al., 1984; Montanini et al., 1988; Stanczyk, 2006). In men and women, non-invasive salivary measures largely confirm parallel diurnal fluctuations in androgenic steroids in serum (Mezzullo et al., 2017). A similar picture emerges in a study (see Figures 2a,b in Guignard et al., 1980) in which T, DHT, Δ4, DHT were monitored over a 24 h interval. Peaks occurred around the time of awakening and early morning and the amplitude of the Δ4 rhythms was higher than that of T and DHT.

##### Estrogens

There are three main circulating estrogens: E1, E2, estriol (E3). E1 is chiefly adrenal derived and is the main form of estrogen for men and postmenopausal women; E2 produced by the ovary, is the most potent estrogen; E3 is synthesized primarily by the placenta during pregnancy. To the best of our knowledge, there have been only two experiments on daily rhythms of E2 levels in female rats or mice. In one study of female rats a repeated daily pattern of serum E2 across the days of the estrous cycle was not detected (Kalra and Kalra, 1974). In the second study a steady increase in E2 levels occurred from diestrus to the time of estrus and ovulation, but no daily pattern was seen (Smith et al., 1975).

In women, the daily pattern of plasma E2 is generally monomodal but the timing of peak plasma levels changes with age, stage of the menstrual cycle, and pregnancy, and also entails inter-individual differences. At puberty in girls, the peak occurs in the early afternoon (Boyar et al., 1976). In adults, plasma levels differ across the menstrual cycle: peak times occur at ∼08:22 h in the menstrual phase and at ∼02:46 h in the late follicular phase cycle (Bao et al., 2003). In the late luteal stage there is an obvious circadian pattern in E2 (Carandente et al., 1989). In postmenstrual women the daytime level of plasma E2 and other steroids is damped (Lønning et al., 1989). In a thorough study of pregnant women that examined the daily rhythms in plasma E1, E2, E3 and CORT in eleven women at 34–35 weeks’ gestation by radioimmunoassay (RIA) sampled at 30–60-min intervals over 24 h, CORT peaked at ∼07:30 h and reached a trough at ∼02:30 h as expected (Patrick et al., 1979). Relevant here, a daily fluctuation was not detected in E2, while E1 and E3 peaked between 10:00 h and 11:30 h.

In men, both the adrenal and testes are sources of circulating E2. Abnormal concentrations of plasma E2 is a feature of many different diseases (reviewed in Table 1 by De Ronde et al., 2003). Experimental studies of the daily pattern of serum E2 present a mixed picture. In one study, the acrophase of plasma E2 appears during the late afternoon, with a large individual difference in the peak level (Juneja et al., 1991). In contrast, in another RIA study that measured E2 every 30 min for 25 h in four males, plasma E2 was constant throughout the day, although E1 appeared to be higher during wake time than sleep (Leymarie et al., 1974). One contributor to differences among studies may be the effect of factors that are hard to control in humans such as stress, food and exercise (Figure 1) on the aromatase enzyme, as these factors are hard to control in studies of humans.

##### Progesterone

In both rodents and humans there is a daily rhythm of circulating P with systematic variation during the ovulatory cycle. In rats, the trough of serum P occurs at mid-day (inactive period) during diestrus, proestrus and estrus with peak levels in the afternoon between 16:00 h and 23:00 h (lights on 05:00 h to 19:00 h) (Kalra and Kalra, 1974). Serum P amplitude is lowest on the day of estrus and then increases progressively until proestrus. In a more recent study aimed at understanding the effects of obesity on daily profiles of circulating P (among other hormones), the steroid was measured at days 15 and 21 of gestation using liquid chromatography-tandem mass spectrometry (Crew et al., 2016). The results indicate a nocturnal decline in P in maternal plasma in control animals, consistent with the earlier findings. Rhythmicity of plasma P at diestrus was lost after ADX in female rats, pointing to an adrenal origin of the hormone at this stage of the cycle. However, at proestrus the peak level of P was five times higher than that of diestrus and also sustained rhythmicity after ADX, pointing to an ovarian source of the hormone at this stage of the cycle (Bailey, 1987).

In male rats, the daily peak of serum P occurred in the early night, with a morning trough, and this pattern was sustained after castration (Kalra and Kalra, 1977). Neither castration nor ADX alone completely abolished P rhythmicity in plasma, consistent with the dual source of this hormone. In a more recent study (aimed at understanding effects of stress), there was no statistically significant effect of time of day on plasma P concentrations in control rats (sampled hourly, lights on: 06:00 h), although visual inspection indicated a trend for a small rise at ∼19:30 h (Hueston and Deak, 2014). Corticosterone (CORT, the primary glucocorticoids in rodents) concentrations for these animals showed the expected daily rhythmicity, indicating good control of experimental conditions. In conclusion, P levels do not show substantial circadian variation in males.

In women, shifts in P levels during the menstrual cycle are evident. The acrophase of plasma P is in the morning during the follicular stage, at midnight during the early luteal stage and in the evening during the late luteal stage (Veldhuis et al., 1988; Carandente et al., 1989; Rahman et al., 2019). Though group data suggest a diurnal pattern of circulating P, there are substantial individual differences (Veldhuis et al., 1988; Carandente et al., 1989). Not all women have a detectable circadian rhythm across the entire menstrual cycle and the variation of peak time can be as broad as 7 h even in subjects studied in the mid-luteal stage. In men, the peak of plasma P appears at 04:00 h and the nadir is at 24:00 h (Opstad, 1994).

Systemic changes in development and external factors affect P levels. In humans of both sexes, salivary P peaks in the morning and declines throughout the day (Gröschl et al., 2003). In aging humans, the daily variation in serum P is maintained in elderly females but not in elderly males (Nicolau et al., 1984). The time of the year may influence the peak and trough time of plasma P. In autumn, for women during the mid-luteal phase, the peak, which is around 07:00 h, time is fairly consistent across subjects and the trough time is around 22:00 h. However, in the spring, there are large individual differences in the circadian pattern of plasma P; the acrophase expands from 07:00 to 20:00 h and the nadir ranges between 03:00 and 15:00 h (Kottler et al., 1989). In addition to age and seasons, stress is another widely studied factor influencing circulating P’s circadian rhythm.

#### Adrenal Hormones

##### Glucocorticoids

The adrenal glands are the sole source of circulating corticoids (Timmermans et al., 2019) unlike sex hormones which derive from multiple sources. Diurnal rhythms in plasma CORT are very well documented. In mice the CORT rhythm is under circadian control, shown by its persistence in conditions of DD and in parallel measures in fecal and plasma samples (Oster et al., 2006b). Peak levels occur at the transition from the inactive phase to the active phase of each day, such that in nocturnal rodents, the peak time occurs at around the time of lights off while in humans, it occurs at around the time of lights on (reviewed in Van Cauter et al., 1996; Spencer and Deak, 2017). This daily pattern is maintained throughout the ovulatory cycle, but in the estrus phase (mice), around the time of ovulation, plasma CORT levels are elevated both in day and night (Nichols and Chevins, 1981).

There are systematic changes in development. One month after birth, the daily rhythm of circulating CORT begins to develop in infants, with no apparent sex differences (Gröschl et al., 2003). Premenopausal women, however, have a lower morning peak than men of the same age range (Van Cauter et al., 1996). With aging circadian rhythmicity is retained in both men and women, but the amplitude is lowered while the mean plasma CORT is increased (Sjöberg et al., 1979; Van Cauter et al., 1996).

##### Mineralocorticoids

Aldosterone is synthesized by the adrenal glands and the peak appears at the transition from the inactive phase to the active phase and then gradually declines throughout the active phase (Ryoyu et al., 1984; Portaluppi et al., 1990). Plasma aldosterone secretion is synchronized with that of CORT in human and rats (Hilfenhaus, 1976; Ryoyu et al., 1984; Cugini et al., 1992). With old age (∼80 years), there is a decrease in plasma aldosterone levels (Cugini et al., 1984) and also there is a consistent decrease in circadian amplitude in women but not in men.

#### Summary

In summary, the overall pattern that emerges from this survey of plasma steroid rhythms is that under steady state conditions, circulating corticoids have readily detectable and consistent diurnal plasma fluctuations. At baseline corticoids have a ∼20-fold peak-trough difference. In contrast, sex hormones, which originate from multiple sources and whose sources and synthesis change with age and stage of the menstrual cycle, display more variable and complex daily variations in the systemic circulation. There is good evidence for substantial differences among individuals, differences between the sexes, strain differences within animal species, seasonal modulation, and changes in development. A report analyzing group data provides pragmatic guidelines suggesting that the time of day that measurements are made contributes markedly to variation among samples for cholesterol, CORT, DHEAS, P, and T, but not to those of E2 (van Kerkhof et al., 2015). We next consider mechanisms whereby the CTS modulate glandular steroid production.

## Circadian Regulation of Steroidogenesis

**FIGURE 4:**
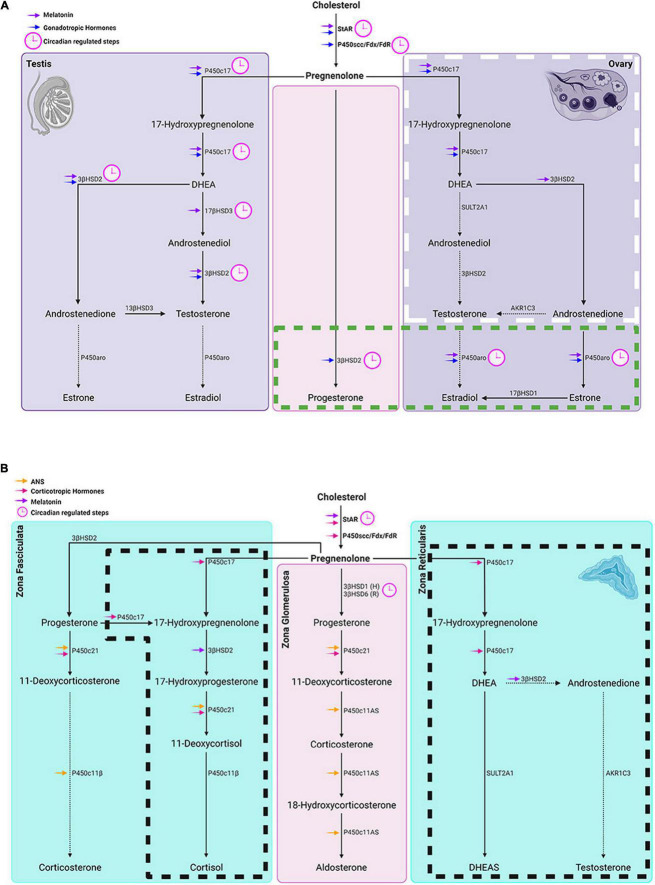
Circadian timing system regulation of gonadal and adrenal steroidogenesis. **(A)** Left and right panels show steroidogenic pathways in the testes and ovaries respectively while the middle panel represents steroidogenic pathways common to both glands. In the ovary, the box with white long-dashed border refers to thecal cell steroid synthesis and the one with a green short-dashed border indicates granulosa cell steroidogenesis. **Symbols:** Blue arrows = regulated by gonadotropic hormones. **(B)** From left to right panels: steroidogenic pathways in adrenal zona fasciculata, zona glomerulosa and zona reticularis respectively. Boxes delineated with dashed lines are pathways in humans that don’t occur in rats and house mice. **Symbols:** magenta arrows = regulated by corticotropic hormones; orange arrows = regulated by the autonomic system; purple arrows = melatonin regulated steroidogenic molecules; (H) in the middle panel denotes primary subtype of 3βHSD in human zona glomerulosa and (R) denotes the primary 3βHSD in rodents. In both **(A,B)**, pink clockfaces refer to steps under circadian regulation; solid arrows point to primary steroid synthetic pathways in humans; dashed arrows point to a hormone that is a minor product of that pathway.

The sites at which steroids act entail both acute and chronic regulatory pathways. The acute response refers to the fast mobilization of cholesterol transportation involving the activation of steroidogenic acute regulatory protein (StAR). The chronic regulatory process determines the quantity and quality of steroids that can be synthesized. In the chronic regulatory process of steroidogenesis, P450scc encoded by *cytochrome P450 11A1* (*CYP11A1*) is the enzymatic rate-limiting step, converting cholesterol to pregnenolone. Other downstream CYPs and hydroxysteroid dehydrogenases (HSDs) determine which steroids are synthesized. A detailed description of tissue specific steroidogenic pathways is provided in Miller and Auchus (2011). In the sections below, the steps in circadian regulation of the steroid synthesis pathways common to all the steroidogenic glands are considered first, and then those of the testes, ovary, and adrenals are discussed in turn (Figures 4A,B). These sites represent the entry points whereby stimuli such as stress, feeding and exercise (discussed below) can affect hormone production.

### Common Synthetic Pathway: *StAR* and *CYP11A1*

Clock genes play a key role in the common steroidogenic pathway. Both *StAR* and *CYP11A1*, components of the common steroid biosynthetic pathway, are ccgs. The transcription of *StAR* while not *CYP11A1* oscillates in adrenal and gonadal steroidogenic cells *in vivo*. However, for both molecules to reach their normal expression level, an intact cellular clock is necessary. In the testes, it is noteworthy that rhythmicity of *StAR* occurs only in the Leydig cells but is not detected when the testis is studied as a whole organ (Alvarez et al., 2008; Son et al., 2008). The level of *StAR* and *CYP11A1* transcripts in isolated mouse Leydig cells becomes rhythmic after synchronization by dexamethasone treatment (Chen et al., 2017). *StAR* expression is significantly reduced in cultured Leydig cells of *BMAL1* knockout mice and goats but *CYP11A1* is not changed (Alvarez et al., 2008; Xiao et al., 2021). In TM3 mouse Leydig cells *bmal1* knockdown decreased *CYP11A1* mRNA (Ding et al., 2020). In ovarie*s StAR* transcription in the human luteinized granulosa cells can be entrained by T stimulation (Chen et al., 2016). Administration of a Rev-erbα agonist elevated *Per2* amplitude and advanced its phase in mouse granulosa cells and significantly increased StAR expression (Chen et al., 2012). In addition, steroidogenic factor 1-driven-*bmal1*-deletion resulted in significantly decreased *StAR* transcription in the mouse ovary (Liu et al., 2014). In mouse granulosa cells, the oscillation of *CYP11A1* expression is synchronized to *bmal1* (Chu et al., 2019). Silencing *bmal1* decreased the expression of *CYP11A1* in granulosa cells.

In the adrenal gland, the StAR protein is rhythmically expressed (Son et al., 2008). To explore whether adrenal clock proteins are driving rhythms in StAR expression, the investigators used a cell line in which a defective mutation of CLOCK (CLOCKΔ19) was used to disrupt the molecular clock. This manipulation caused decreased StAR transcription. On the other hand, overexpression of CLOCK and BMAL1 in the same cell line increased the level of StAR protein. In contrast to StAR, daily P450scc concentration is constant. In another study, the level of cholesterol associated with P450scc was also not changed over time of day (Brownie et al., 1979). These results show that in the adrenal gland StAR rather than P450scc is a key molecule in the circadian regulation of the common steroidogenic pathway.

The expression of *StAR* and *CYP11A1* are also influenced by gonadotropins and adrenocorticotropins. In the testes, hCG induces a series of clock gene expression in rat Leydig cells, including *Per1*, *Rorb Rev-erba, Rev-erbb*, and *BMAL1*, and also increased *StAR* mRNA level (Baburski et al., 2019). The same study also showed that low levels of circulating LH and T in male rats altered expression levels of clock genes *bmal1, Per2, Cry1, Cry2, Rorα/β, Rev-erbα/β*, decreases the level of *StAR* and *CYP11A1* and eliminates rhythmicity in *CYP11A1*. In rat granulosa cells, LH entrains the oscillation of *StAR* and *CYP11A1* (Chen et al., 2013), and FSH stimulates oscillation of *StAR* (Chen et al., 2012). In the adrenal cortex, ACTH is a strong phase-setting signal (Yoder et al., 2014) and ACTH pulses induce *StAR* and *CYP11A1* in rat adrenal glands (Spiga et al., 2011).

The expression of StAR is also under the diurnal regulation of melatonin in both gonads and adrenal glands [testes (Frungieri et al., 2017), ovaries (Tamura et al., 2009; Rai and Gosh, 2021) and adrenal glands (Minnetti et al., 2020)].

### Testicular Steroidogenic Enzymes

In the testes, in addition to *StAR* and *CYP11A1* of the common synthetic pathway, *3β-HSD*, *CYP17A1* and *17β-HSD* (encoding 3βHSD2, P450c17 and 17βHSD respectively) are also ccgs. In mouse and goat cultured Leydig cells, dexamethasone entrained the circadian variation of *3β-HSD*, *CYP17A* and *17β-HSD* (Chen et al., 2017; Xiao et al., 2021). *bmal1* knockout in goat Leydig cells reduced the expression of *3β-HSD* whereas *bmal1* overexpression increased *17β-HSD* mRNA level. Global knockout *of bmal1* reduced the expression of *3β-HSD*, *CYP17A* and *17β-HSD* in mice (Alvarez et al., 2008). Some testicular steroidogenic genes are also under the regulation of upstream hormonal signals. The *de novo* synthesis of P450c17 in mouse Leydig cells requires cyclic adenosine monophosphate (cAMP) stimulation, which can be induced by the gonadotropins (Payne and Youngblood, 1995). *3βHSD2* has a relatively high basal level without cAMP. However, to reach its maximum expression, it requires cAMP induction. The expression of *CYP17A1* oscillates in the normal rat Leydig cells but its rhythmicity is lost in Leydig cells of rats with insufficient LH and T (Baburski et al., 2019).

Melatonin has an inhibitory effect on testicular steroidogenesis. In the hamster Leydig cells, *3β-HSD* and *17β-HSD* expression was reduced by this hormone (reviewed by Frungieri et al., 2017). Pinealectomy started the oscillation of *3β-HSD* in the rat Leydig cells and this change can be reversed by melatonin supplement (Baburski et al., 2015).

### Ovarian Steroidogenic Enzymes

In the ovary, *CYP19A1* (encodes P450 aromatase) expression has a diurnal rhythm in rat and mouse granulosa cells (Chen et al., 2013; Chu et al., 2019). Silencing *bmal1* in granulosa cells significantly suppressed the expression of *CYP19A1. 3β-HSD* has large variations over 24 h but is not rhythmic in rat granulosa cells (Chen et al., 2013). *Bmal1* siRNA in these cells decreased the level of *3β-HSD.*

The variations of LH and FSH across the reproductive cycle influence the homeostasis among steroidogenic enzymes in the ovary (Niswender et al., 2000). In the transition from follicles to corpora lutea, the expression of P-producing *3β-HSD* is increased, and the estrogen-producing *CYP19A1* and androgen-producing *CYP17A1* are decreased. In the rabbit ovary, the activity of promotor on *CYP19* responsive to cAMP in the luteal cells decreased by 50% compared to granulosa cells (Andrieu et al., 2009). Human chorionic gonadotropin is stimulatory to the expression of *3β-HSD* and *CYP19A1* in granulosa cells. However, unlike *StAR*, the expression pattern of these two genes is not oscillatory in the human granulosa cells (Chen et al., 2017). Low dosage of LH can stimulate *CYP17A1* expression in bovine thecal cells *in vitro* only when they are not pretreated with a high dose of LH. This is a simulation of the pre-ovulatory milieu (Murayama et al., 2012). When they are pretreated with a high dose of LH, low dose of LH no longer stimulates *CYP17A1*, a condition that simulates the post-ovulatory environment of theca cells.

Melatonin also regulates the mRNA level of the enzymes important in ovarian androgen and estrogen synthesis. In porcine antral follicles, melatonin inhibited expression of CYP*17A1* but not *CYP19A1* (reviewed by Tamura et al., 2009; Rai and Gosh, 2021). Pinealectomy increased *CYP17A1* in rat theca interna and interstitial cells and *CYP19A1* in granulosa cells whereas chronic administration of melatonin decreased *CYP17A1* (Maganhin et al., 2014; Lima et al., 2015).

### Adrenal Steroidogenic Enzymes

#### Endogenous Adrenal Clock

As for StAR, circadian oscillation occurs in other steroidogenic genes, namely *17β-HSD7*, *lanosterol synthase*, *StAR-related lipid transfer protein5, farnesyl diphosphate synthetase*, and *24-dehydocholosterol reductase* evidenced in studies of mRNA profiling in adrenal tissue collected from mice held in DD (Oster et al., 2006a). These genes are involved in cholesterol synthesis and intracellular translocation, and steroid metabolism.

#### Suprachiasmatic Nucleus to Adrenocorticotropic Hormone and Melatonin

The SCN modulates the oscillation of steroidogenic gene expression in the adrenal cortex by signaling ACTH and melatonin (Tsang et al., 2014). ACTH potently regulates adrenal cellular clocks and the expression of steroidogenic molecules (Simpson and Waterman, 1988; Yoder et al., 2014). ACTH binding to its receptor leads to increased intracellular cAMP level and activates downstream signaling pathways, including the action of the cAMP responsive element modulator (CREM). In the adrenal glands of wild type mice, CYP17A1 is not expressed due to hypermethylation (Košir et al., 2012). As noted previously, this is why rodent adrenals don’t produce androgens. In *Crem* knockout mice, *CYP17A1* shows hypomethylation leading to upregulation of this enzyme at the time of lights off. Thus, *Crem* knockout induces the diurnal variations of *CYP17A1* but blunts the rhythmicity of *CYP21A1* (encodes P450c21). Finally, melatonin acts as an inhibitory factor in adrenal steroidogenesis, attenuating the expression of *3β-HSD* induced by ACTH (see review by Minnetti et al., 2020).

#### Suprachiasmatic Nucleus to Autonomic Nervous System

In addition to the foregoing classical control of the adrenal gland by ACTH, light induces gene expression in the adrenal gland via a direct SCN-sympathetic nervous system pathway via the splanchnic nerve (Ishida et al., 2005). The response is abolished upon lesioning the SCN. This pathway is the basis of fast induction of Nr4a1 and Nr5a1 expression in response to light pulses in the subjective night. These transcription factors further increase the expression of steroidogenic enzymes *CYP21* (encodes P450c21) and *CYP11B2* (encodes P450 aldosterone synthase). This light-induced pathway results in an increase of plasma CORT levels without activating the HPA. Impressively, the magnitude of the CORT response is dose dependently correlated with the light intensity.

#### Aldosterone

In the adrenal, the expression of *3β-HSD* in aldosterone producing cells of the zona glomerulosa is clock-controlled. In the mouse, this gene encodes HSD3B6 a homologue of HSD3B1 in human zona glomerulosa, which is important in aldosterone synthesis and has been implicated in hypertension. Heterozygous *Per1* mice have reduced levels of *3β-HSD* compared to wild type animals (Richards et al., 2013). Global knockout of *Cry1/2* increases the expression of *3β-HSD* (Doi et al., 2010).

In summary, the foregoing evidence indicates multiple sites of action whereby the CTS impacts steroid synthesis in both gonadal and adrenal glands. As suggested in the summary diagram in Figure 2, a number of behavioral and environmental factors can also influence the synthesis of steroids.

## Differential Afferent Inputs to Peripheral and SCN Clocks

While the SCN serves as a brain clock, it is but one component of a multi-level CTS that underlies daily and circadian rhythmicity. The clocks in various steroidogenic tissues are not uniform in response to afferent signals. There is a tremendous amount of evidence showing that various behaviors, such as eating patterns, stress and exercise, impact circadian clocks in the periphery. Here we point to some of the effects of the foregoing cues on peripheral and SCN clocks.

### Feeding

Cues related to feeding are thought to be among the most potent signals for peripheral clocks. Important in the present context, glucocorticoids are thought to play a key role in synchronization of the rest of the body to photoperiod and food availability (Balsalobre et al., 2000). For example, restricting food access to the light phase in nocturnal rodents reverses the timing of peak and trough expression of the clock genes *per1*, *per2*, and *bmal1* in the adrenal gland (Girotti et al., 2009). Restricting feeding to daytime also reverses the diurnal peak and trough of *StAR* in the adrenal glands (Girotti et al., 2009; Chung et al., 2017). Interestingly, under these conditions CORT secretion has a bimodal pattern with one peak related to the adrenal clock and the other driven by the SCN, suggesting that the food induced peak is independent of the SCN and that the actions of the central clock on the adrenal glands remain undisturbed. In addition to the effects of time of eating, specific food components also influence the expression of glandular clock genes, steroidogenic molecules, and the concentration of circulating steroids.

As for the food components, in mouse testes, a high fat diet significantly reduced the level of *bmal1, clock, per2, cry2* at the time of lights on but not at the time of lights off. Also, this diet initiated a difference between the light on and light off time in expression of *SF-1, StAR, CYP11A1, 3β-HSD3, 17β-HSD17* and in plasma T, while no difference was seen in *ad libitum* fed controls (Wang et al., 2018). In another study, a high fat diet abolished the bimodal secretion pattern of T shown in control rats fed a normal diet, and the average level of plasma T was suppressed (Cano et al., 2008). In this experiment, the high fat diet blunted the daily variations of CORT but increased its mean value by 61%.

In female mice, a high fat diet significantly decreased *bmal1* expression in the ovary near the end of dark phase (Yokoyama et al., 2020). *StAR* and *CYP11A1* were downregulated at the beginning of light phase and *CYP11B1* was downregulated at the beginning of dark phase. These changes eventually led to increased daytime levels of caecal CORT. In comparison, in the same study a high fat, high salt diet group had decreased expression of *cry2*. There was a trend toward reduction of *StAR* by this diet, and this was accompanied by a significant, bimodal pattern of plasma CORT. In a study of developmental effects, a high fat diet impacted cellular clocks in hormone production by steroidogenic cells. Thus, both maternal and/or post-weaning high fat diet in pregnant rats increased *per1*, *per2* and *per3* mRNA expression in the ovary of the female offspring, and elevated plasma E2 (Lin et al., 2017).

An important consideration here is that while there are numerous examples demonstrating powerful effects of feeding in modulating peripheral clocks, some studies indicate that the SCN is resistant to these cues (Balsalobre et al., 2000; Hara et al., 2001; Wakamatsu et al., 2001). But this generalization is not universally true. Feeding-related cues also act on the central clock. Thus, while rhythms of per1 and per2 mRNA in the SCN were not altered by restricted feeding, *bmal1* expression was lower in the morning and increased in the evening compared to control animals – a significant feeding condition by time interaction (Girotti et al., 2009). Restricted feeding entrains PER2 oscillation in the SCN of rats whose rhythm were disrupted by constant light (Lamont et al., 2005). The genetic background of the subjects may be a factor underlying the effects of feeding. Thus, the oscillation of per1, *per2* and *bmal1* in the SCN can be entrained by restricted feeding in CS mice but not C57BL/6J mice under DD (Abe et al., 2007). In another study suggesting strain differences in SCN sensitivity to food cues, four lines of house mice were examined (Castillo et al., 2004). After long term exposure to scheduled feeding under DD, PER2 oscillation in the SCN was synchronized to food anticipatory activity in 100% of the mice in one line, and only some of the mice in the other three lines. In summary, decoupling time of eating and activity and altering diet components produces phase-shifting in glandular clocks and steroidogenesis. Further exploration of work on how eating and what is eaten effectively change diurnal rhythms can be found in Lewis et al. (2020).

### Stress

Stressors are powerful factors regulating diurnal variations in steroidogenesis. In humans, a combination of stressors including strenuous exercise, sleep and energy deprivation for 5 days leads to a significant decrease in the mean values of plasma T, Δ4, DHEAS, progesterone and CORT, and blunts their diurnal rhythms (Opstad, 1994). However, the effect of stress on peripheral clocks, on steroidogenesis, and on the SCN differs depending on the type and duration of the stressor, and on the time of day of stress administration. Acute stressors including those lasting minutes to hours and those lasting several days, modulate the expression of peripheral clocks and steroidogenic molecules. In contrast, chronic stressors lasting weeks to months sometimes lead to habituation (reviewed in Koch et al., 2017; Tahara et al., 2017). For example, acute stress has a very rapid impact on adrenal steroidogenesis. After 1 h of restraint, increased StAR mRNA was detected in rat zona fasciculata, accompanied by increased CORT secretion (Liu et al., 2013). Restraint stress applied for 15 min advanced adrenal PER2::LUC oscillation by 2 h (Engeland et al., 2016). In another study, 2 h restraint starting during the mid-day didn’t shift adrenal PER2::LUC expression, but repeating the regimen for 7 days did do so (Stagl et al., 2018). Finally, 14 days of subordination stress also led to advanced PER2::LUC in the adrenal glands (Razzoli et al., 2014).

Acute stress also disrupts the cellular clock and steroidogenesis in gonads and other tissues. For example, acute immobilization stress increases *bmal1*, *per1/2* expression, thereby shifting the peak of these clock mRNA expression toward the end of the stress; this stressor also decreases *Rev-erbα*; at the same time, it blunts the rhythmicity of *Rev-erbβ* and *cry1* (Medar et al., 2021). In fact, a variety of steroidogenic genes in the Leydig cells, including *StAR*, *CYP11A1*, 3β-*HSD1/2*, *CYP17A* are decreased by this stress regimen. All these changes lead to decreased mesor and blunted rhythm of T, revealing the powerful effect of short-term stress in regulating daily steroid production. For the ovary, there is much research on the impact of stress on ovarian steroid biosynthesis and reproductive function (reviewed by Whirledge and Cidlowski, 2010; Toufexis et al., 2014) and on how circadian clock disruption influences the ovarian steroidogenesis, ovulation, fertility and fecundity (reviewed by Sellix, 2015). To our knowledge however, there is no data available on how the stressors modulate the ovarian clock thereby impacting ovarian steroidogenesis.

The effects of chronic stressors differ from those of short-term stress. CORT secretion is significantly reduced under chronic stress compared to the shorter duration regimens (Tahara et al., 2015). Although potent in reshaping the peripheral clocks and steroid synthesis, it is generally reported that stress does not appear to influence the rhythm of SCN PER::LUC (Takahashi et al., 2001, 2013; Razzoli et al., 2014; Tahara et al., 2015). However, this is not a universal finding. Thus, 19 days of social defeat stress in the dark phase on mice leads to decreased PER2 positive neurons in the SCN, as well as resulting in decreased *per2* and *cry1* in the adrenal glands (Bartlang et al., 2014). Also, 4 weeks of unpredictable stress (a combination of diverse stressors, including restraint, tilt, forced swimming etc.) caused a significant decrease in the peak value of PER2 expression in the SCN about 3 h before lights are off (Jiang et al., 2011).

### Physical Exercise

Like food and stress, exercise also functions as a powerful non-photic synchronizer, both in the periphery and in the SCN. In the periphery, both voluntary and forced exercise affect the clocks and steroidogenesis and increases plasma CORT (Pieper et al., 1995; Kanaley et al., 2001). In a voluntary exercise paradigm in mice that had free access to a running wheel, the peak of PER2::LUC was delayed in liver and adrenal glands compared to animals without access to a wheel (Schroeder et al., 2012). The same study showed that scheduled exercise, implemented by limiting access to a wheel to either early or late night (active) phase delayed liver PER2::LUC oscillation, whereas the adrenal PER2::LUC was delayed only by the early night access. In contrast, forced exercise in the inactive (light) phase significantly advanced PER2::LUC rhythm and *per2* and *bmal1* mRNA expression in lung, liver and adrenal gland, and also advanced CORT secretion (Wolff and Esser, 2012; Sasaki et al., 2016). Regular voluntary exercise over weeks steadily increased the average level of CORT (Otawa et al., 2007). This effect was probably caused by increased peak value of *StAR* expression in the adrenal glands.

In the SCN, in contrast to the periphery, voluntary exercise has a stronger effect than forced exercise. Spontaneous activity suppressed SCN firing rate and increased the amplitude of the electrical activity, whereas forced behavior increased the firing rate (van Oosterhout et al., 2012). In a voluntary running paradigm, limiting the time of access to a wheel influences SCN responses. In mice held in an LD cycle, limiting access to a running wheel in the early night led to reduced PER2::LUC amplitude in the SCN (Schroeder et al., 2012). In contrast, limiting access to a running wheel during the light phase did not affect PER2::LUC oscillation in the SCN (Wolff and Esser, 2012). Manipulating access to wheel running also entrains locomotor rhythms in VIP and/or VIP receptor knockout mice. In these genetically modified animals, scheduled exercise under DD increased the amplitude of PER2::LUC oscillation in the ventral SCN, an effect opposite to that seen in the wild type animals (Power et al., 2010; Hughes et al., 2021). These results indicate the potential of voluntary exercise in shaping the SCN clock is phase dependent.

Forced exercise however, produced by confining subjects to a treadmill, did not affect the SCN (Wolff and Esser, 2012; Sasaki et al., 2016). The reason of the differential effects of voluntary and forced exercise on the SCN may be that forced exercise is a psychological stressor, pointing to different mechanisms underlying the entraining potential of the two types of exercise. Taken together, the examples show that non-photic entraining signals including feeding, stress and exercise are potent in shaping rhythmicity in peripheral clocks and steroid synthesis. Steroid hormones, especially glucocorticoids, modulated by non-photic cues relay circadian signals to extra-SCN brain regions and peripheral tissues (review in Spencer et al., 2018). Given that feeding, chronic stress, and voluntary exercise can each promote steroid secretion and can also alter SCN cellular clocks under certain circumstances, the results provoke the question of what might be the mechanism whereby these afferent cues act on the SCN. As we discuss next, the possibility that steroid receptors in the SCN are a mediating mechanism merits examination.

## Steroid Receptors in the Suprachiasmatic Nucleus

**FIGURE 5:**
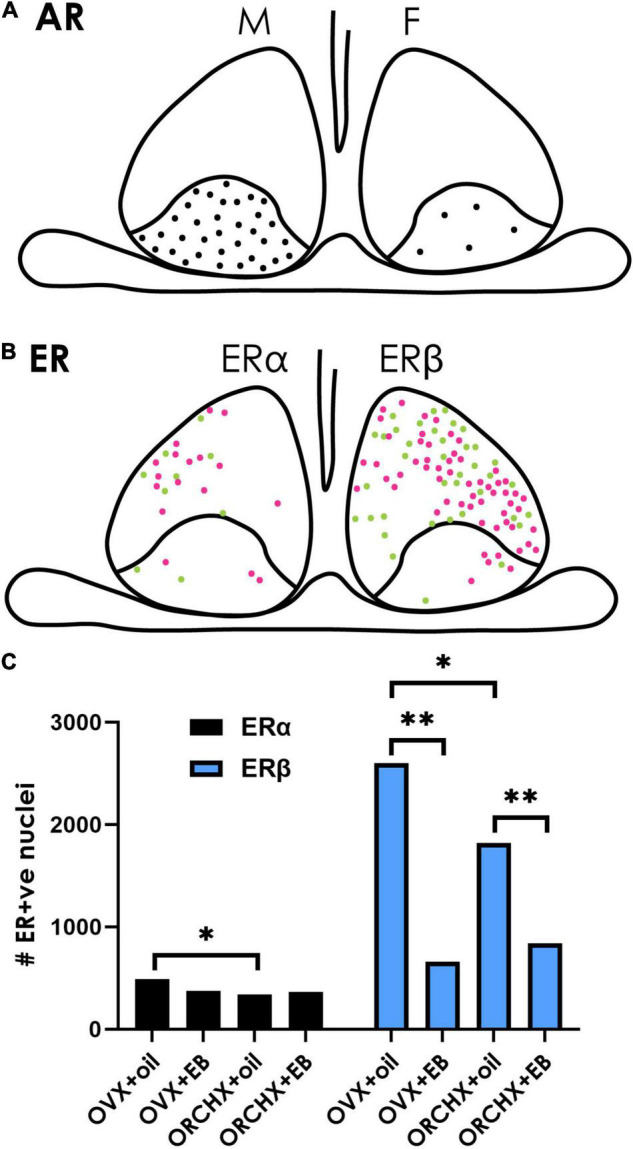
Sex hormone receptors in the SCN. **(A)** The cartoon depicts the localization of androgen receptors (ARs) in males and females. ARs are restricted to the SCN core, and their density is greater in males (left) than in females (right). Data from Karatsoreos et al. (2007). **(B)** ERs are mostly localized in the shell SCN in female mice. There are fewer ERα-(left) than Erβ- (right) positive neurons. Magenta vs. green dots represent higher vs. lower staining intensity. **(C)** Quantification of ER in the mouse SCN after gonadectomy with/without estrogen supplement indicates that ovariectomized (OVX) females have higher ERα/β than orchidectomized (ORCHX) males. Images adapted from Vida et al. (2008), with permission from John Wiley and Sons. **Symbols:** Black bars show counts of ERα. Blue bars show counts of ERβ. Single asterisk refers to significant difference between ovariectomized females and oil treated males and ovariectomized and oil treated females. Double asterisks refer to difference between oil and β-estradiol-3-benzoate (EB) treated males or females.

**FIGURE 6:**
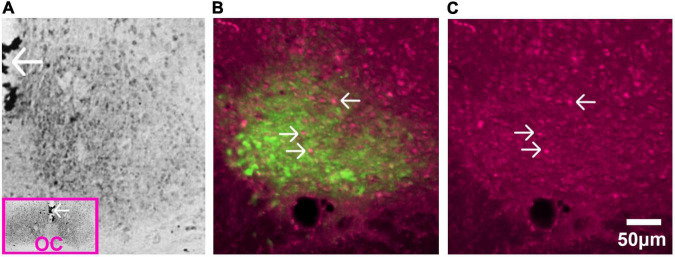
Glucocorticoid receptors in SCN of adult rat. **(A)** Immunostaining of GR detected by avidin-biotin-peroxidase complex in the adult rat. The pink box in the inset is a low magnification image of the section and the arrows pointing to the third ventricle provide orientation. Abbreviation: optic chiasm (OC). Image adapted from Rosenfeld et al. (1988), with permission from Elsevier. **(B)** AVP staining identifies the shell region of the SCN. Image shows fluorescent double label for GR (magenta) and AVP (green) in the adult rat SCN. **(C)** Single label fluorescent stain for GR alone is shown in **(C)**. In both **(B,C)**, arrows point to GR expressing neurons.

There are a striking number of sites at which hormonal and neural signals act on gonadal and adrenal glands with the end result that fluctuations occur in systemically circulating steroid hormones. These hormones reach the SCN and its steroid receptors (Figures 5, 6). Though presence of steroid receptors and density of steroid receptors has been reported in a number of studies (summarized in Table 3), their function and mechanisms of action are virtually unknown. Given the ultradian and circadian fluctuations in steroids, it is important to note that the time course of changes in receptor expression can be very rapid in response to a hormone. Systemically administered T can change receptor expression within 15 min (Freeman et al., 1995).

**TABLE 3:** Density of steroid receptors in the SCN.

Receptor	Species	Sex	Age	Intensity	Localization	Assay	References
AR	Human	M	Adult	++	?	IHC	Kruijver and Swaab, 2002
		F	Adult	+	?	IHC	
	Rat	M	Adult	+	Shell	IHC	Jahan et al., 2015
	Mouse	M	Adult	+++	Core	IHC	Wang et al., 2007
		F	Adult	+	Core	IHC, WB	Iwahana et al., 2008
	Ferret	M	Adult	+++	Homogeneous	IHC	Kashon et al., 1996
	Baboon	F	Fetal	++	?	Autoradiography	Handa et al., 1988
		M+F	Fetal	+	?	IHC	Wu et al., 1995
	Goat	M	Adult	+++	?	IHC	Maejima et al., 2009
ERα	Human	M	Adult	++	?	IHC	Kruijver and Swaab, 2002
		F	Adult	+++	?	IHC	
	Mouse	ORCHX M	Adult	+	Shell		Vida et al., 2008
		OVX F	Adult	++	Shell		
	Rats	OVX F	Adult	−	?	ISH	Shughrue et al., 1997b
		M+F	PND0	+	N/A	ICC	Su et al., 2001
	Plains vizcacha	F	Adult	++	?	IHC	Inserra et al., 2017
ERβ	Human	M	Adult	+	?	IHC	Kruijver and Swaab, 2002
		F	Adult	++	?	IHC	
	Mouse	ORCHX M	Adult	+	Shell	IHC	Vida et al., 2008
		OVX F	Adult	+++	Shell	IHC	
	Rats	OVX F	Adult	+	?	ISH	Shughrue et al., 1997b
		M+F	PND0-19	+	?	ISH	Cao et al., 2014
		M+F	PND0	++	N/A	ICC	Su et al., 2001
	Sheep	M	Adult	+	?	ISH	Hileman et al., 1999
PR	Human	M	Adult	++	?	IHC	Kruijver and Swaab, 2002
		F	Adult	++	?	IHC	
	Rats	M	Adult	−	N/A	IHC	Auger and De Vries, 2002
	Ginea pigs	F	Adult	−	N/A	IHC	Blaustein et al., 1988
	Rabbits	F	Adult	−	N/A	IHC	Caba et al., 2003
	Japanese macaques	F	Pubertal	−	N/A	IHC	Goldsmith et al., 1997
	Crab-eating macaques	M+F	Adult+ pubertal	−	N/A	IHC	Bethea et al., 1992
GR	Mouse	?	Adult	–	N/A	ISH	Balsalobre et al., 2000
	Rats	M	PND2	+++	?	IHC	Rosenfeld et al., 1988
			PND8	+++			
			PND12	++			
			PND16	++			
			PND20	+			
			Adult	+			
		M	Adult	+	?	IHC	Ahima and Harlan, 1990
		M	Adult	+	?	IHC	Cintra et al., 1994b
		M	Adult	+	?	ISH	Su et al., 2015
MR	Rats	M	Adult	+	?	IHC	Ahima et al., 1991
		M	Adult	+	?	ISH	Su et al., 2015

*AR, androgen receptors; ER, estrogen receptor; F, female; GR, glucocorticoid receptor; ICC, immunocytochemictry; IHC, immunohistochemistry; ISH, in situ hybridization; M, male; MR, mineralocorticoid receptor; N/A, not applicable; ORCHX, orchidectomized; OVX, ovariectomized; PND, postnatal day; WD, western blot; ?, not reported; −, not detected; +, weak expression; ++, intermediate expression; +++, strong expression.*

### Androgen Receptors

Androgen receptors (ARs) have been identified in the SCN of many species, including humans, mice, rats, ferrets, goats, and baboons (Handa et al., 1988; Wu et al., 1995; Kashon et al., 1996; Fernández-Guasti et al., 2000; Wang et al., 2007; Iwahana et al., 2008; Maejima et al., 2009; Jahan et al., 2015), pointing to the potentially broad generality of receptor-mediated mechanisms. Gonadectomy reduces AR expression in mice (Karatsoreos et al., 2007), and systematically administrated T restores AR expression in a concentration dependent manner (Butler et al., 2012). ARs in mice are located in gastrin-releasing peptide containing neurons lying in the core region of the SCN (Figure 5A). They express FOS after a light pulse indicating that they are retinorecipient. Importantly, gonadectomy reduces the FOS response after a phase-shifting light pulse, whereas androgen replacement restores the FOS response to that seen in intact animals. The results highlight the fact that retinal input and the androgenic steroid act on the same neurons within the SCN (Karatsoreos and Silver, 2007).

There is a sex difference in AR expression in the SCN with more robust expression in men vs. women and in male vs. female mice (Fernández-Guasti et al., 2000; Wang et al., 2007). This difference is attributable to adult circulating T as the sex difference disappears following castration of male mice and is reinstated with T administration (Iwahana et al., 2008). In females, administration of T by implants of silastic capsules in gonadectomized mice produces a male-like patterns of SCN AR expression. Finally, in the four core genotype mouse model (gonadal males with either XX or XY chromosomes and gonadal females with either XX or XY chromosomes) gonadal males have more AR than gonadal females, indicating that the sex difference in AR expression in the SCN is independent of sex chromosomes (Kuljis et al., 2013).

The functional significance of ARs in the SCN has been demonstrated by direct intracranial implants of T. Following gonadectomy, the free-running period of locomotor activity is lengthened in male mice and testosterone treatment reverses these effects (Daan et al., 1975). Importantly, unilateral intracranial T implants in gonadectomized mice reinstate both intact-male-typical locomotor activity and ipsilateral SCN AR expression (Model et al., 2015). The results indicate that androgens acting directly within the SCN are sufficient to support male-typical circadian locomotor rhythms. The mechanism of how a change in AR expression results in altered behavior remains unexplored.

### Estrogen Receptors

ERα and/or ERβ receptor have been detected in the SCN in many species, including humans, rats, mice, plains vizcacha, sheep (Shughrue et al., 1997a,b; Hileman et al., 1999; Su et al., 2001; Kruijver and Swaab, 2002; Vida et al., 2008; Cao et al., 2014; Inserra et al., 2017). The expression of both ERα and ERβ has been delineated in mice (Figure 5B; Vida et al., 2008). In cultured SCN cells and astrocytes of neonatal rats ERα and ERβ are colocalized (Su et al., 2001). In young and adult, but not old rats, ERβ mRNA in the SCN has a diurnal rhythm, peaking around 03:00 h in animals held in a 14:10 LD cycle with lights on at 04:00 h (Wilson et al., 2002). As in the case of T, circulating estrogens affect receptor expression. A single injection of β-estradiol-3benzoate reduced ER-β expression in SCN of gonadectomized male and female mice, though ER-α was not affected by estrogen administration (Vida et al., 2008; Figure 5C). In both young and old OVX rats, ER-β mRNA expression was decreased in SCN after implantation of E2-bearing capsules (Shima et al., 2003; Yamaguchi and Yuri, 2014).

There are sex differences in SCN ER (Figure 5C). About 25.5% of cells in female mouse SCN and 16% in male show ERβ-ir neurons in the SCN respectively, approximately 2% of which co-express AVP (Vida et al., 2008). In humans, females have more ERα in the SCN than males (Figure 6 in Kruijver and Swaab, 2002). ERβ demonstrates five- to sixfold higher immunoreactivity than ERα. The quantification of ERα and ERβ also demonstrated a dimorphic pattern, with nuclear ERα significantly higher in women’s SCN.

Significant effects of estrogen on circadian period, measured by locomotor activity, are very well documented. For example, ovariectomy increases free-running period and E2 replacement shortens free-running period in hamsters, rats, and mice. The period-shortening effects of E2 can be mimicked by agonists of both the ERα and ERβ, with ERα agonists effective at lower doses (Joye and Evans, 2021).

Systemic application of E2 alters the expression of *Cry1* and *Cry2* mRNA (Nakamura et al., 2001), affects FOS-IR (Gibbs et al., 1990; Peterfi et al., 2004) and *Per2* mRNA (Nakamura et al., 2005), alters neurotransmitter and transporter activity (Hery et al., 1984; Krajnak et al., 2003), and changes responsiveness to Ach and 5-HT (Kow and Pfaff, 1984). Given that these studies involve systemic hormone application, they do not probe the site of action and may be due to afferent input from extra-SCN target sites (Mong et al., 2011; Hatcher et al., 2020).

Direct bath application of E2 to the SCN increase the spontaneous firing frequency and depolarized cell membrane of SCN neurons in whole-cell patch clamp recordings (Fatehi and Fatehi-Hassanabad, 2008). These responses were thought to be receptor mediated as all these effects were abolished by pretreatment with the E2 receptor antagonist, ICI 182780. However, *in vitro* exposure of SCN tissue from OVX rats exposed to high physiological levels of E2 did not significantly alter the period of *per1-luc* expression (Murphy et al., 2013). E2 treatment didn’t affect PER2::LUC either (Sellix et al., 2006; Nakamura et al., 2008). These results suggest that either E2 does not act directly on the SCN or that E2 does act on the SCN but in a manner not detected by this measure.

### Progesterone Receptors

In men and women, PRs are found in SCN neurons, in both cellular nuclei and cytoplasm, with no significant sex differences (Figures 4C,D in Kruijver and Swaab, 2002). Little or no PR is reported in the SCN in rats, guinea pigs, rabbits and monkeys (Blaustein et al., 1988; Bethea et al., 1992; Goldsmith et al., 1997; Auger and De Vries, 2002; Caba et al., 2003; Murphy et al., 2013). Very high concentrations of P and P+E2 lengthened the period of *Per1-luc* in SCN explants, while physiological levels of the steroids were not effective (Blaustein et al., 1988; Bethea et al., 1992; Goldsmith et al., 1997; Auger and De Vries, 2002; Caba et al., 2003; Murphy et al., 2013), leading the authors to conclude that at physiological levels ovarian steroids do not directly affect the timing of *Per1-luc* expression in SCN neurons despite the presence of E2 and P4 receptors in SCN pacemaker neurons.

### Glucocorticoid Receptors

The two corticoid receptors, glucocorticoid receptors (GR) and mineralocorticoid receptors (MR), are functionally differentiated by their responses to circulating glucocorticoids, with MR mainly participating in the baseline activity of glucocorticoids and GR regulating acute responses to stress (Kloet and Reul, 1985; Spencer and Deak, 2017). CORT receptors in the SCN change markedly in the postnatal period. In neonatal rats, the expression of GR is high during the first week after birth. GR immunoactivity gradually declines, and by PND20, only low density of GR-ir was detected in this brain area, comparable to the expression level of adult rats (Rosenfeld et al., 1988, 1993; Ahima and Harlan, 1990; Cintra et al., 1994b). There is a correlation during development in rats between the rise of circulating CORT and the decrease of glucocorticoid receptors in the SCN (Henning, 1978; Rosenfeld et al., 1988).

In a widely cited study, it was reported that there is no significantly detectable GR mRNA in the SCN of adult mice (Balsalobre et al., 2000). The authors postulated that an SCN devoid of corticosteroids receptors provides a mechanism to resist the impacts of sudden hormonal changes in circadian rhythms resulting from stress. However, several studies report the presence of GR protein or mRNA in adult (Rosenfeld et al., 1988; Cintra et al., 1994a,b; Morimoto et al., 1996; Figure 6). Additionally, real-time polymerase chain reaction analysis indicates that GR is present at low levels and is upregulated after ADX (Su et al., 2015). The available evidence is sparse; in some studies of GR and MR distribution in adult brain, the SCN is not mentioned (Aronsson et al., 1988; Sutanto et al., 1988; Sousa et al., 1989), while in other work, GR is studied only in young animals (Yi et al., 1994).

As in the case of systemically administered estrogen, many studies point to corticoid effects on the SCN though direct vs. indirect sites of action cannot be determined.

Glucocorticoids modulate light entrainment of a molecular clock in the SCN and also affect locomotor activities, indicating the stress hormone may participate in regulating the circadian timing system through the central clock (Sage et al., 2004; Wang et al., 2012). Systemic treatment with dexamethasone changed the level and/or rhythmicity of AVP and VIP mRNA expression in the SCN (Larsen et al., 1994). Consistent with this result, in post-mortem material from humans treated with glucocorticoids, the AVP mRNA was substantially lower than in patients who died of diseases where glucocorticoids was not administered (Liu et al., 2006), suggesting an effect of corticoid treatment. Systemic injections of dexamethasone upregulated Fos-ir and Jun-ir in the SCN (Briski et al., 1997). ADX upregulated *cry2* mRNA expression in the rat SCN (Su et al., 2015) and abolished its oscillation. ADX also reduced the immunostaining of glial fibrillary acidic protein in the ventral SCN and CORT supplement reversed this effect (Maurel et al., 2000). Dexamethasone shifted the phase of PER2::LUC in fetal mouse SCN explants (Čečmanová et al., 2019) pointing to a direct effect of the steroid on the SCN in early development. In the adults, ADX had no impact on the phase of *per1-Luc* rhythms in the SCN (Wang et al., 2012). Exogenous administration of CORT induced higher AVP expression in the SCN only during the early morning indicating that stress hormones may feedback the SCN in a narrow time window (Larsen et al., 1994).

### Mineralocorticoid Receptors

Low density immunostaining of MR (Ahima et al., 1991) and its mRNA expression (Su et al., 2015) has been detected in the adult rat SCN. MR mRNA level does not vary across day. Similar to effects on GR, ADX appears to elevate *MR* expression in the rat SCN. However, to our knowledge, there is no understanding of the function on the MR in the SCN.

### Conclusion

The evidence for the existence of steroid receptors in the SCN is robust. The evidence clearly supports the conclusion that there are receptors of androgens estrogens and corticoids in the SCN. There is also evidence that circulating steroids have direct effects on SCN function. The hypothesis that emerges from this evidence is that steroids act on their cognate receptors in the brain clock and alter its sensitivity to afferent input, its oscillation and/or its output. Surprisingly little work has been done to explore mechanisms of action and function of SCN steroid receptors. Nevertheless, the foregoing review suggests that SCN steroid receptors have functions. Behavioral and physiological factors related to feeding, sexercise and stress each affect hormone secretion. Though few in number, there are studies indicating that each of these factors can alter SCN activity, either by altering sensitivity to light, or changing the timing or expression level of specific genes or proteins.

Consider the possibility that hormonal inputs to the SCN are specialized, and that they share some but not all properties with afferent photic input. The SCN clock responds to regularly recurring photic signals at specific times of the day. If the SCN’s responses to CORT have similar properties to those of SCN responses to light, then we must anticipate that the GR mediated responses will occur to regularly recurrent signals, and that the efficacy of those signals will be time of day dependent. Additionally, in the adult, the retinorecipient region of the SCN encompasses many more neurons than those bearing GR. As a consequence, it may require a substantial and recurrent hormonal cue for the few GR neurons to effectively alter the overall oscillation of the SCN. Importantly the time course of changes in receptor expression can be very rapid in response to a hormone. For example, systemically administered T can change receptor expression within 15 min (Freeman et al., 1995).

Cortisol or corticosterone is an especially attractive and most ignored candidate as an important SCN acting signal. This is the least noisy of the rhythms in hormones, a desirable feature in a timing mechanism. Plasma corticoid levels have a reliable high amplitude daily rhythm with a distinct peak with a 20-fold change as a function of time of day (Spencer and Deak, 2017). They provide a clear time stamp as they have a relatively narrow peak at the onset of the active phase in both diurnal and nocturnal species. Stress, feeding and exercise-mediated alterations of the daily pattern are readily discerned on this steady baseline. CORT blood levels rise within several minutes after stressor onset, typically reach peak levels within 30 min, and can return to pre-stress levels within 60 min after discrete stressor termination. These transients rest atop a stable baseline.

Corticoids acting on GR are known to be effective in entraining clock gene expression in peripheral and extra-SCN brain tissue. Interestingly while GR are thought to be ubiquitous throughout the body, they are considered virtually absent in the adult SCN (Balsalobre et al., 2000). In fact, there are GR receptors in the adult SCN, though they are sparsely distributed (Figure 6).

Given the sparse distribution and small numbers of GRs, these neurons are not likely to produce a response on the oscillator population as a whole, unless the cue is robust and repeats. There is a lack of experiments testing the possibility that the SCN responds to regularly recurring hormonal cues which might explain the function of these receptors in the SCN. One problem is that there are many potential responses that might be relevant. For example, a steroid might act by increasing or decreasing the amplitude of rhythms by changing the period of oscillations, altering sensitivity to phase shifts, lowering the peak or raising the trough of oscillations, changing the signal to noise ratio, altering phase relationships among oscillators, responding to phase-setting signals, etc. (reviewed in Silver and Balsam, 2010). And of course, it may be that one clock gene or protein is affected but another is not. For example, in studies of effects of daylength on the SCN, it appears that Per1 and Per2 play differential roles in photoperiodic responses (Yoshikawa et al., 2017).

## Summary and Overview

When describing circadian rhythms, we and many others start with the conceptualization of the SCN as a master clock, but *in vivo*, this master is much informed by “non-masters.” The non-master clocks focused on here are those of steroid synthesizing glands and the hormones they secrete into the systemic circulation. To the best of our knowledge, we know almost nothing about how these afferent signals alter SCN activity.

Ultradian and diurnal/circadian rhythmic variations and transient changes in circulating steroid hormones, their plasma levels, their sites of action, their effects at various sites, etc. occur simultaneously and are constantly changing over time. Systemically circulating steroids reach all clock cells of the body and provide cues that are both regularly recurring and transient. Largely ignored is the evidence for androgen, estrogen and corticoid receptors in the SCN. The hypothesis that emerges from these considerations is that steroids act on cognate receptors in the SCN and most investigators would agree that if a receptor is present, it must have a function.

One problem is that there are many potential responses that might be relevant, even if only a single measure is considered. For example, a steroid might act by increasing or decreasing the amplitude of rhythms by changing the period of oscillations, altering sensitivity to phase shifts lowering the peak or raising the trough of oscillations, changing the signal to noise ratio, altering phase relationships among oscillators, responding to phase-setting signals, etc. (reviewed in Silver and Balsam, 2010).

Optimal alignment of oscillators throughout the body is a key aspect of health. Misalignment of circadian oscillators is associated with severe health disturbances and increased the risk of a variety of diseases, including cardiac, metabolic, mental diseases and cancer (Baron and Reid, 2014). For example, non-oscillatory, high level of CORT interrupts adipose metabolism, leading to excess fat accumulation (Bahrami-Nejad et al., 2018). Such results shed light on understanding the chronobiological basis of close relation between chronic stress and obesity. However, little is known of how disrupted hormonal rhythms will directly influence the brain’s clock. Only for T has a direct effect on the SCN been demonstrated (Model et al., 2015). The work summarized above indicates that same SCN neurons that bear T receptors also receive direct RHT input and that activation of these receptors by direct, local T implants in GDX mice is sufficient to restore circadian locomotor activity to that seen in intact animals. However, the mechanism involved is unknown. In fact, there is a general lack of research on the impact and function of steroid receptors in the SCN. Research on the contribution of circulating hormones to afferent input to the SCN will deepen the knowledge about the importance of alignment of oscillators in maintaining overall health.

## Author Contributions

YY and RS wrote the manuscript and approved the final version. Both authors contributed to the article and approved the submitted version.

## Conflict of Interest

The authors declare that the research was conducted in the absence of any commercial or financial relationships that could be construed as a potential conflict of interest.

## Publisher’s Note

All claims expressed in this article are solely those of the authors and do not necessarily represent those of their affiliated organizations, or those of the publisher, the editors and the reviewers. Any product that may be evaluated in this article, or claim that may be made by its manufacturer, is not guaranteed or endorsed by the publisher.

## Abbreviations

ACTH: adrenocorticotropic hormone
ADX: adrenalectomy
AR: androgen receptor
ARC: arcuate nucleus
Δ 4: androstenedione
AVPV: anteroventral periventricular nucleus
AVP: arginine vasopressin
ANS: autonomic nervous system
cAMP: cyclic adenosine monophosphate
CORT: cortisol or corticosterone
CREM: cAMP responsive element modulator
CTS: circadian timing system
ccg: clock-controlled gene
CRH: corticotropin releasing hormone
CYP: cytochrome P450
DD: constant darkness
DHEA: dehydroepiandrosterone
DHEAS: dehydroepiandrosterone sulfate
DHT: dihydrotestosterone
E2: estradiol
E3: estriol
ER: estrogen receptor
E1: estrone
FSH: follicle stimulating hormone
GnRH: gonadotropin releasing hormone
hCG: human chorionic gonadotropin
HSD: hydroxysteroid dehydrogenase
HPA: hypothalamic-pituitary-adrenal axis
HPG: hypothalamic-pituitary-gonadal axis
Kp: kisspeptin
LD: light:dark
LH: luteinizing hormone
OVX: ovariectomy
POA: preoptic area
P: progesterone
PR: progesterone receptor
RIA: radioimmunoassay
RHT: retinohypothalamic tract
RORE: retinoic acid-related orphan receptor response element
SCN: suprachiasmatic nucleus
StAR: steroidogenic acute regulatory protein
T: testosterone
VIP: vasoactive intestinal polypeptide.
